# Contrasting Maternal, Paternal, and Biparental Ancestry of Populations From the Caribbean Coast of Colombia

**DOI:** 10.1002/ajpa.70242

**Published:** 2026-04-09

**Authors:** Masinda Nguidi, Christina Amory, Catarina Xavier, Gabriela Huber, Filipa Simão, Beatriz Martinez, Luis Caraballo, Leonor Gusmão, Walther Parson

**Affiliations:** ^1^ DNA Diagnostic Laboratory (LDD), State University of Rio de Janeiro (UERJ) Rio de Janeiro Brazil; ^2^ Institute of Legal Medicine, Medical University of Innsbruck Innsbruck Austria; ^3^ i3S Institute for Research and Innovation in Health, University of Porto Porto Portugal; ^4^ Department of Chemistry and Forensic Science Towson University Towson Maryland USA; ^5^ Institute for Immunological Research, University of Cartagena Cartagena Colombia; ^6^ Forensic Science Program, The Pennsylvania State University University Park Pennsylvania USA

**Keywords:** ancestry informative markers, bolivar, NGS, population admixture, population substructure

## Abstract

**Objectives:**

The current Caribbean Colombian population is the result of migration processes and admixture that occurred throughout the country's history. The aim of this study was to investigate genetic ancestry gradients throughout the Caribbean territory, with a higher Native American ancestry expected in inland areas compared to coastal cities. Simultaneously, since the genotyping methodology used included markers of forensic relevance for predicting population of origin and pigmentary phenotypic characteristics, this study also tested the main tools commonly applied in forensic contexts.

**Materials and Methods:**

Uniparental and biparental genetic ancestries of individuals from the Bolívar Department, in the Caribbean Colombian region, were investigated. A total of 64 samples were collected from Coast, Center, and Inland regions. Genotyping was performed with a newly developed *PCR‐based targeted* MPS tool called COMBO, targeting over 1000 polymorphisms along mtDNA, Y chromosome, and autosomes.

**Results and Discussion:**

The Coast and Center regions showed similar ancestry profiles. The Inland region displayed a contrasting pattern, being mainly composed of Native American maternal lineages and European paternal lineages. Statistically significant differences in mtDNA and AIM‐SNP compositions were only found between the Coast and Inland regions, absent for the Y chromosome. The results obtained regarding the prediction of eye, hair, and skin color were as expected based on the ancestry profile of the population. Biogeographic ancestry prediction tools have presented challenges in assigning individuals to the American metapopulation, highlighting the need for better reference datasets from South America, given the great heterogeneity in the admixture patterns they present.

## 1 Introduction

Signs of the first human occupancy of the Colombian territory date back around 15,000 years ago. Native Americans arrived from North America through the Isthmus of Panama, spreading in two directions: into the Andes (west) and into the Amazon (east) (Guhl 2010). As in most parts of the Americas, the genetic composition of Colombia reflects successive waves of migration into the territory: the initial settlement of the Americas by Indigenous peoples, the European colonization (16th–19th centuries), and the forced arrival of enslaved Africans. As the main gateway for European colonizers arriving in Colombia, Bolívar represents an interesting target for study, since it includes the urban region of Cartagena, where the most important port in Colombia for the slave trade was located. European colonization led to the establishment of cities and capitals near port areas to facilitate trade and communications. The exponential growth of urban centers influenced the distribution of the local populations, as most Europeans and enslaved Africans settled in these urban hubs. As a result, many Indigenous groups retreated to remote (inland) areas, forming refuge settlements. Most Indigenous populations in the Caribbean eventually dispersed throughout the region, often forming alliances with other population groups, such as the *palenques* (Ángel 1998; Jaramillo Uribe 1964; Solano and Flórez 2007).

The *palenques* emerged in Colombia as a form of refuge and pursuit of freedom for Afro‐descendants who had been enslaved during the colonial period. The largest and most significant of these settlements is *San Basilio de Palenque* (Schwegler et al. 2017), located about 50 km from the port areas and major centers of Cartagena de Indias city. While these communities were primarily composed of formerly enslaved individuals, alliances were formed with Indigenous groups who joined *palenques* (also known as *maroon* communities), also escaping from European colonialism (Ángel 1998; Jaramillo Uribe 1964). Based on the historical data described above, the existence of a population substructure in the Caribbean region associated with geography would be expected, with gradients of Native American and African ancestry dependent on the distance from the coastal region, near the main ports of arrival of Europeans and Africans. While it is easy to infer an increasing gradient of Native ancestry toward the interior of the region, the distribution pattern of African ancestry becomes more difficult to predict. Upon arrival in Colombia, enslaved Africans were concentrated in the most urbanized regions, which suggests a lower African ancestry in populations far from the ports of arrival. However, the formation of *palenques* in inland areas and interactions with Native groups may have preserved the African heritage in regions of more difficult access.

Therefore, the main aim of this study was to elucidate the genetic structure of a region of the Caribbean that comprises the main port of arrival for Africans trafficked to Colombia. In order to enlighten possible geographic gradients of Native American and African ancestry previously mentioned, this study included samples from individuals born in the Coast, Center, and Inland regions of the Department of Bolívar. The ancestry of these three regions was analyzed using a previously described panel, named COMBO (Amory et al. 2025), which comprises markers located on the Y chromosome (Y‐Chr), mitochondrial DNA (mtDNA), and autosomes. The use of uniparental markers also allowed the investigation of population dynamics by crossing information from lineages of maternal and paternal origin.

The newly developed Massive Parallel Sequencing (MPS) panel COMBO is a *PCR‐based targeted* sequencing methodology that allows sequencing 781 Y‐SNPs (Thermo Fisher Scientific Technical note; https://assets.thermofisher.com/TFS‐Assets/GSD/Technical‐Notes/hid_ion_ampliseq_visage_technical_note.pdf), the whole mitogenome (Strobl et al. 2018), and 115 autosomal ancestry informative (AIM) SNPs (Xavier et al. 2020) simultaneously in a single reaction. Since this panel was developed for forensic applications, a set of 41 SNPs known as HIrisPlex‐System (Chaitanya et al. 2018) was also incorporated to enable the prediction of externally visible traits. The forensic application of the COMBO panel, specifically for predicting eye, hair, and skin color, as well as determining biogeographical ancestry (BGA) in admixed populations was evaluated when applied to individuals from the Caribbean.

## 2 Materials and Methods

### 2.1 Extraction, Quantification, and Amplification of the Targets

**FIGURE 1:**
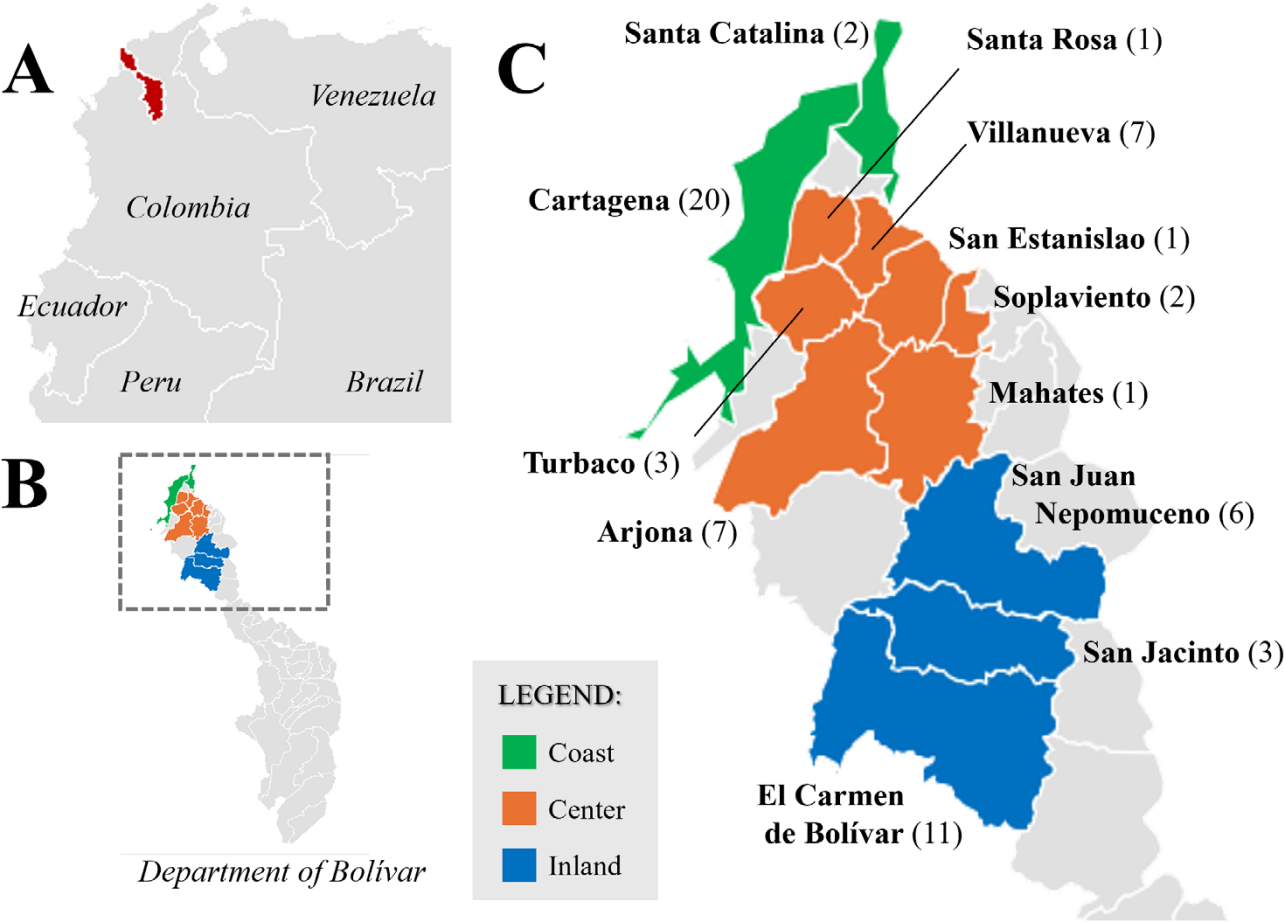
Maps representing: (A) the location of Bolivar (in red) inside Colombia; (B) the location of the 12 studied municipalities in the Coastal region of the Department of Bolívar; and (C) the number of studied samples per municipality.

A total of 64 unrelated male samples from the Coast, Center, and Inland Caribbean regions of the Colombian Department of Bolívar were analyzed in the present study (Figure 1). The study was approved by the institutional ethics committee—*Comité de Ética en Investigaciones*—of the University of Cartagena (approval number: Acta no 40 from 2012; updated in Acta no 108 from 2018). All the participants included in this study donated whole blood samples through a campaign promoted by the universities at the respective hospitals (Cartagena University Hospital, Hospital Universitario del Caribe at Carmen de Bolívar) and medical clinics in San Juan Nepomuceno. The samples were collected under informed consent. Throughout this study, samples were coded and treated anonymously, and only information regarding sex and department of birth was retained. Participants were randomly selected from the general population and did not represent specific vulnerable groups or closed communities. It is important to emphasize that the donors were not selected based on ethnic or community criteria; therefore, they come from diverse backgrounds and do not represent any specific community that could have been engaged in the research process.

DNA was extracted from total blood by the salting out method (Miller et al. 1988). Samples were quantified by real‐time PCR using a tetraplex system, which can detect the presence of inhibitors and targets autosomal, two mtDNA targets, and one internal positive control (Xavier et al. 2019). According to the nuclear DNA concentration, all the samples were normalized to 3 ng/μL with RNAse free water.

The COMBO targets were amplified in two separate PCRs, both including the primers for all 934 autosomal and Y‐chromosomal SNPs, but a different pool of mtDNA primers (Pool 1 and Pool 2), as previously described by Amory et al. (2025). Both PCRs were performed in a 20 μL final volume, with 1 μL of DNA at 3 ng/μL, 5 μL of H_2_O, 4 μL of 5× Ion AmpliSeq HiFi Master Mix and 10 μL of Primer Mix 1 or 2 (1.25 μL Precision ID mtDNA Whole Genome Panel Pool 1 or 2 + 4 μL 5× VIS‐BT‐Primer + 4.475 μL 5× Ion AmpliSeq HID Y‐SNP Research Panel v1 + 0.275 μL H_2_O). The amplification comprised an initial step for 2 min at 99°C; followed by 22 cycles of (1) denaturation for 15 s at 99°C and (2) annealing and extension for 4 min at 60°C; and a last hold at 10°C.

### 2.2 Library Preparation and Sequencing

A partial digestion of the primers and phosphorylation of the 5′ end of the PCR amplicons was performed with the addition of 2 μL FuPa reagent (Thermo Fisher Scientific, Waltham, USA) to each sample. The reaction was performed in a 22 μL volume, for 10 min at 50°C, 10 min at 55°C, and 20 min at 60°C. The ligation of adapters and barcodes was carried out as described in the application guide (Applied Biosystems, publication number MAN0017770). The libraries were purified with magnetic beads using the Agencourt AMPure XP Reagent (Beckman Coulter Life Sciences), following manufacturer's protocol, and quantified with the TaqMan Quantitation kit (Thermo Fisher Scientific) in a QuantStudio 5 (Thermo Fisher Scientific). The libraries were diluted to 30 pM. An emulsion phase on IonSphere (ISP) and loading of the libraries onto Ion 540 Chips were made automatically by the Ion Chef System (Thermo Fisher Scientific). A total of 11 samples were combined per run/chip. Sequencing was performed in an Ion S5 Semiconductor Sequencer (Thermo Fisher Scientific).

### 2.3 Data Analysis

The Torrent Suite Software v5.10.0 (Thermo Fisher Scientific) was used to check the performance of the pooling and run, and to align FastQ raw data to the reference genome GRCh37—hg19, producing BAM/BAI files. The Converge software was used to align mtDNA to the Revised Cambridge Reference Sequence (rCRS; Andrews et al. 1999) from the Precision ID mtDNA panel that contains an appendix of 80 nucleotides after position 16,569. Mitogenomes were analyzed using the Integrative Genomics Viewer—IGV software (Robinson et al. 2023; http://software.broadinstitute.org/software/igv/), and EMPOP tools (Parson and Dür 2007; Parson et al. 2014; Zimmermann et al. 2011; Dür et al. 2021; https://empop.online) were used for quality control of the results. The software IGV was also used to confirm genotypes and alleles from the Y panel. The Y‐SNP haplogroups were determined using Yleaf (Ralf et al. 2018) and PhyloImput software (https://zehrakoksal.com/Y_software.html).

Arlequin ver. 3.5.1.2 software (Excoffier and Lischer 2010) was used to perform population differentiation analyses between the three Caribbean regions, by means of genetic distances (*F*_ST_), non‐differentiation probabilities, and Analysis of Molecular Variance (AMOVA) and to estimate diversity parameters.

To integrate our results in the context of South America, phylogenetic analyses were performed for the subset of African and Native American mtDNA lineages found in our samples, and incorporating published data from South American and African mitogenomes. Given the low representation of the African genetic pool in many South American countries (namely, Peru, Paraguay, Argentina, and Ecuador), a comparative analysis based on haplogroup frequencies showed results strongly influenced by the small sample sizes, making them uninformative.

A similar phylogenetic analysis based on Y‐Chr haplogroups was not possible, since most of our haplogroups were European and lacked haplotypic data. For the pool of European haplogroups, a population differentiation analysis was performed between Caribbean and European populations from Portugal (Beleza et al. 2006), Iberia (Adams et al. 2008), France (Bekada et al. 2013), Italy (Boattini et al. 2013), Germany (Rębała et al. 2012), and Lebanon (Zalloua 2008) by means of genetic distances (*F*_ST_). For the Native and African Y‐Chr lineages, the most likely origin was inferred based solely on their continental distribution reported in the literature due to the low representation of these lineages in our dataset for a meaningful genetic distance analysis.

Full mitogenome data from South American and African populations were compiled from the following publications: De Saint Pierre et al. (2012), Barbieri et al. (2014); 1000 Genomes Project Consortium et al. (2015), Brandini et al. (2018), Simão et al. (2019), García et al. (2021). The mtDNA haplotypes were converted into sequences and aligned using Haplosearch (Fregel and Delgado 2011), after excluding indels at positions 16030–16193, 16194–309, 310–315, 316–522, 525–573, and 574–576. The mtPhyl (software Tool for Human mtDNA Analysis and Phylogeny Reconstruction, developed by Eltsov & Volodko in 2011) was used to construct phylogenetic trees of African and Native American haplogroups.

The 115 AIM‐SNP profiles were analyzed using the GenoGeographer (*apps.math.aau.dk/aims/*) to test the software's predictions on the population of origin using the “VISAGE Basic tool”. This tool uses available reference meta‐populations from Africa, Europe, America, East Asia, the Middle East, and South Asia (Mogensen et al. 2020). The software STRUCTURE (Pritchard et al. 2000) was used to estimate admixture proportions of the input samples using the “Admixture Model” and “Allele frequencies correlated” settings. A total of 400,000 Markov Chain Monte Carlo (MCMC) iterations were computed (disregarding the first 200,000 interactions), and three population clusters were assumed (K = 3) (African, European, and Native American). Reference population data used in STRUCTURE analyses were retrieved from the 1000 Genomes database (1000 Genomes Project Consortium et al. 2015), and consisted of 108 Africans, 99 Europeans, and 85 Native Americans. The STATISTICA data analysis software system, ver. 8.0 (TIBCO Software Inc. Palo Alto, CA, USA) was used to perform Principal Component Analysis (PCA) based on AIM‐SNPs profiles.

The HIrisPlex‐S eye, hair, and skin color DNA phenotyping webtool (https://hirisplex.erasmusmc.nl/) was used to predict phenotypic traits.

## 3 Results

The BAM and CSV files automatically generated from sequencing with the COMBO tool were used in the analyses and quality control. For mtDNA, the acceptance criterion had a minimum limit of 10 reads per base, with complete haplotypes obtained for 56 samples. The remaining seven samples showed partial profiles at nucleotide positions 249–298, 250–383, 4618–4710, 8343–8415, 10578–10649, 10780–10866, 10776–10869, 12388–12454, 12391–12438, and 13906–13988 (see Table S2). For autosomal and Y‐chromosome SNP data, loci with low genotype quality (GQ = 0) and/or that did not present a minimum coverage of five reads were disregarded. Seven of the 115 AIM‐SNPs could not be genotyped in at least one sample, with the following error rates: 1.6% for rs7736783, rs1398461, and rs5757362; 3.1% for rs4737753 and rs2605361; and 15.6% for rs12498138 and rs2196051. For this set of markers, 45 samples presented complete profiles, 13 samples failed in only one marker, 4 samples in 2 markers, and 2 failed in 3 markers. For HIrisPlex‐S, results comprising 41 SNPs were fully obtained in five of the 63 samples. Failures were observed in the markers rs10756819 (BNC2 gene), rs1470608, and rs1545397 (OCA2 gene), with error rates of 90%, 81%, and 76.2%, respectively. For the Y chromosome, a failure in the P33_1 marker (rs24987987) was observed in all samples. After disregarding this SNP, 14 samples resulted in complete profiles. Of the 780 Y‐SNPs, 78 failed in at least one sample, with error rates ranging from 1.6% to 49.2% (mean = 19.3%).

After this filtering, one sample with less than 98% coverage of the complete mitogenome was eliminated from subsequent mtDNA analyses resulting in a final number of 63 samples. For the Y chromosome, 63 samples were considered for final analyses, with one sample being eliminated for having 55 Y‐SNPs with a low GQ value. The complete set of 64 samples was used in ancestry and phenotypic inferences.

### 3.1 Ancestry Informative Autosomal Markers

The ancestry proportions of each individual based on the 115 AIM‐SNP profiles are available in Table S1.

One sample from this study presented a “G” allele at rs11778591, with a heterozygous profile (G: 50%|C: 50%). The rs11778591*G was not reported in the 1000 Genomes Project Phase 3 or in the NCBI ALFA populations (see the Ensembl database https://www.ensembl.org/Homo_sapiens/Info/Index). This allele was described only in the gnomAD genomes v4.1 database, with a frequency of 46 in 152,040 alleles. All samples harboring this rare allele belong to admixed populations, namely Admixed American and African American. It is worth noting that in this study, the rs11778591*G was found in an individual with near 50% African ancestry, supporting a possible African origin of this allele.

**FIGURE 2:**
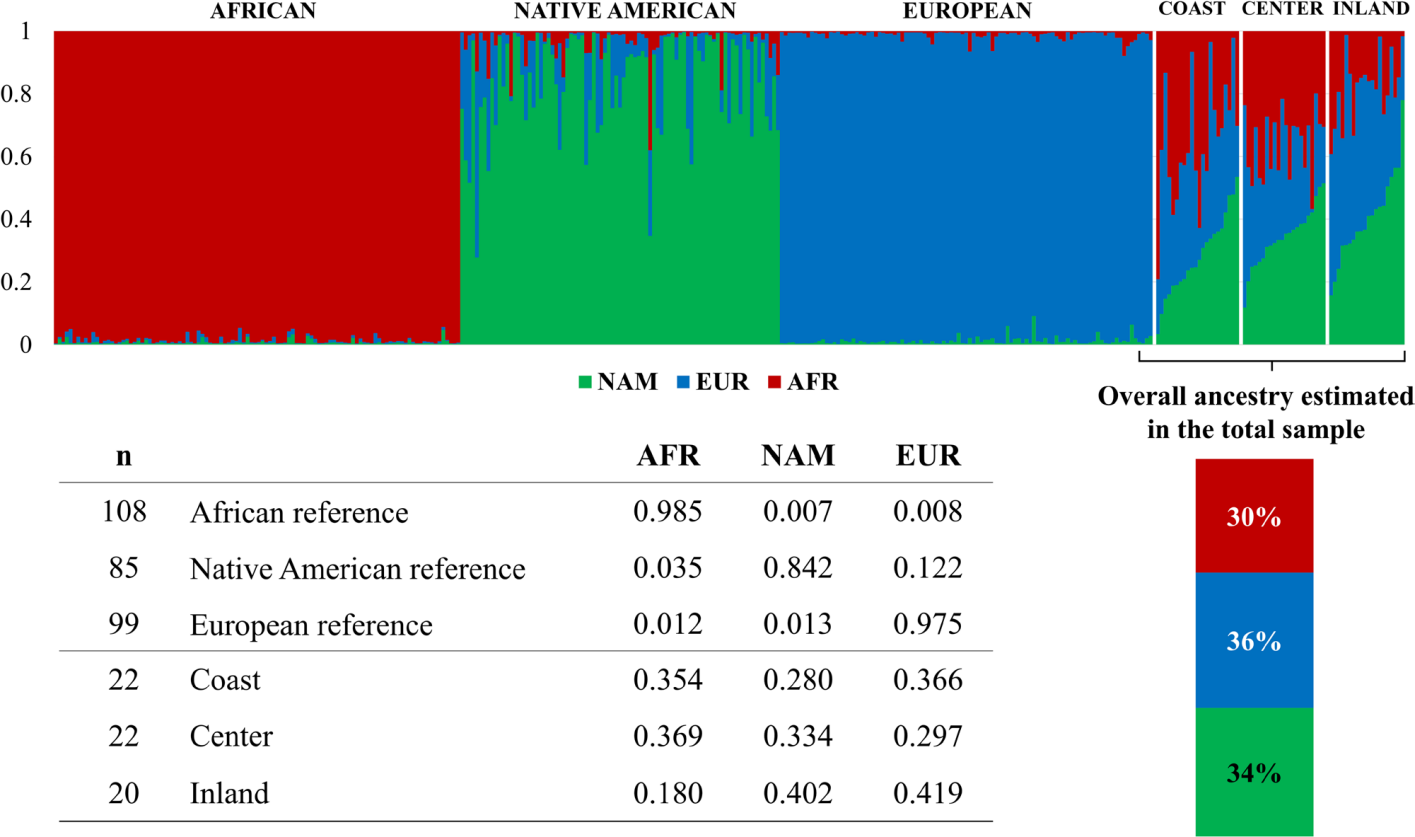
Ancestry proportions obtained with STRUCTURE for the Caribbean Colombian samples, considering three source populations (k = 3). In the table, the mean values of ancestry contributions from Africa (AFR), Native America (NAM), and Europe (EUR) are presented.

The proportions of African (AFR), European (EUR), and Native American (NAM) ancestries obtained for the three regions separately, as well as for the total sample, are depicted in Figure 2.

The results show a highly admixed population, with an average contribution of ~30% of each continental ancestry in the overall population sample (Figure 2). Nonetheless, ancestry proportions are highly variable among individuals, and slight differences can be observed between the three regions, with an increase in Native American ancestry detected toward the Inland region.

**FIGURE 3:**
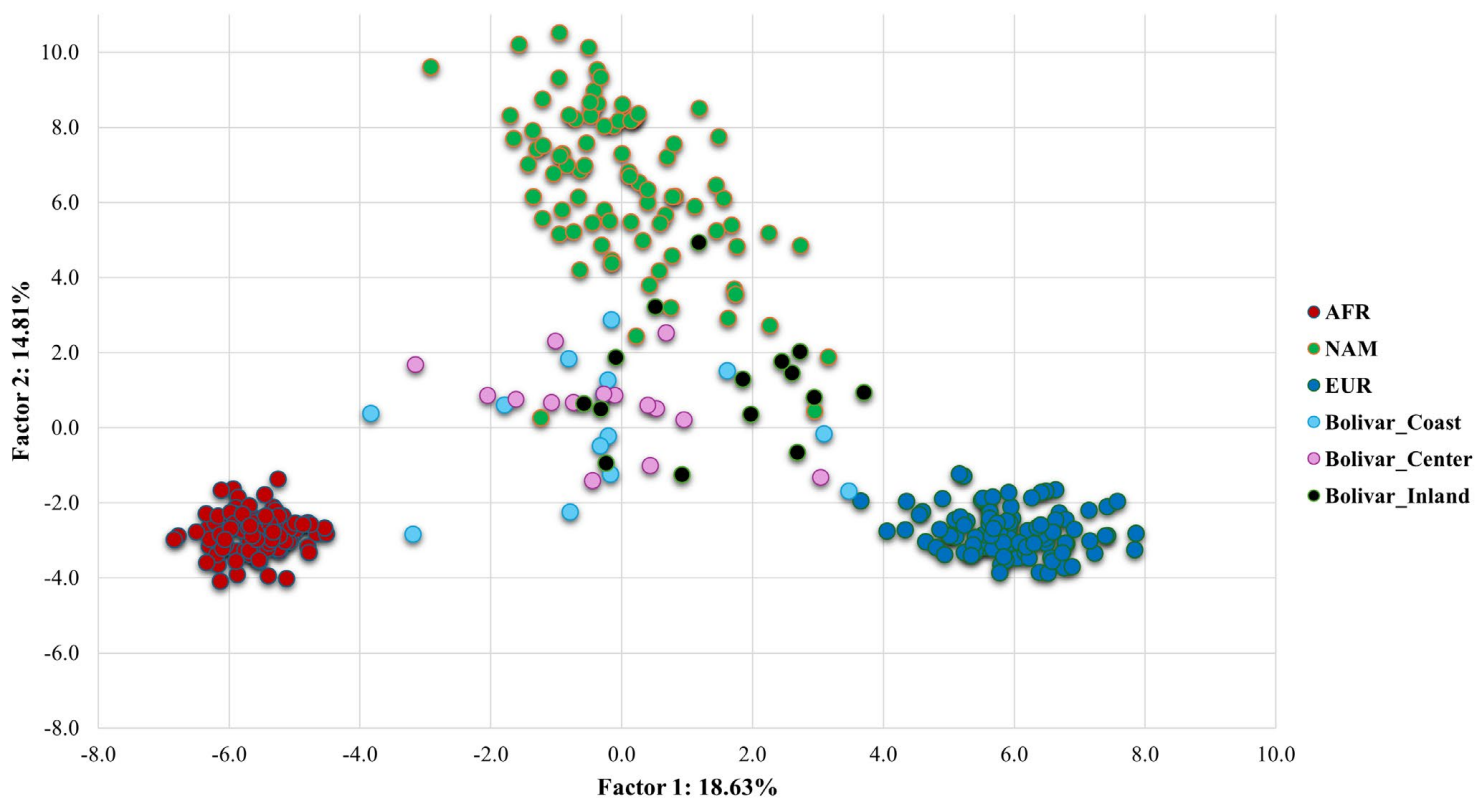
Principal component analysis (PCA) plot performed with 115 AIM‐SNPs complete profiles.

PCA was conducted to investigate genetic distances between our samples and those from reference populations from Africa, Europe, and America (Figure 3). Corroborating the admixture estimates obtained with STRUCTURE, the Colombian samples are widely spread between the EUR, AFR, and NAM reference clusters. Nonetheless, different distribution patterns can be observed among individuals from the three regions. Individuals from the Coast and Center regions are positioned in the center of the triangle formed by the reference populations. Those from the coastal region are more dispersed, reflecting greater variability within this region. Individuals from the Inland region are further away from the African reference cluster, evidencing greater Native American and European ancestries.

Based on the 115 AIM‐SNP profiles, genetic distances and non‐differentiation *p*‐values were calculated between the three studied regions from the Bolívar Department. Statistically significant pairwise *F*_ST_ values were obtained between (i) Coast and Inland (*F*_ST_ = 0.0145; *p* = 0.001) and (ii) Center and Inland regions (*F*_ST_ = 0.0134; *p* = 0.001). The difference between Coast and Center regions (*F*_ST_ = 0.0015; *p* = 0.45) was not statistically significant.

### 3.2 Mitochondrial DNA

The mtDNA haplotypes of the samples included in this study are available in Table S2. A high haplotype diversity was found (HD = 0.9954 ± 0.0041), with 56 different haplotypes, from which 51 were unique.

A total of 30 different haplogroups were found in the general sample, with lineages traced to African ancestry being more diverse than Native American ones (Table 1). No European maternal lineages were detected.

**TABLE 1:** mtDNA haplogroup composition and frequencies in the Coast, Center, and Inland regions, with the proportions of haplogroups assigned to an African (AFR) or Native American (NAM) origin.

Haplogroups	Coast	Center	Inland
NAM origin	57.1%	54.5%	90.0%
A2 + (64)	3	3	5
A2 + (64) +@153	—	—	1
A2af1a1	—	1	—
A2al	—	2	—
A2w	—	—	1
B2b	2	1	—
B2d	5	4	3
C1b	—	1	—
C1c	1	—	—
C1c3	—	—	4
C1d1	—	—	1
D1	1	—	3
AFR origin	42.9%	45.5%	10.0%
L0a1a1	—	—	1
L1c2a1b	—	1	—
L1c3a1b	1	—	—
L1c3c	1	—	—
L2a1a2	1	2	—
L2a1c4a	1	—	—
L2a1c5	1	—	—
L2a1n	1	1	—
L2b1a3	—	—	1
L2d1a	—	1	—
L3d1a1a	—	1	—
L3d1b1	1	—	—
L3e1d1	1	—	—
L3e1e1	1	—	—
L3e2b	—	1	—
L3e4a	—	1	—
L3f1b1a	—	1	—
L4b2b1	—	1	—

The Coast and Center regions showed similar percentages for Native American lineages as well as African ones. In the Inland region, 90% of the maternal lineages were of Native American origin and 10% were African. Based on mtDNA haplotypes, genetic distance (*F*_ST_) analyses were performed between the three regions. A statistically significant distance was found in the comparison between Coast and Inland regions (*F*_ST_ = 0.043; *p* = 0.04), but not between Coast and Center (*F*_ST_ = −0.019; *p* = 0.84) or Center and Inland (*F*_ST_ = 0.021; *p* = 0.13) regions. In contrast, when the same analysis was performed based on mtDNA haplogroups, no statistically significant differences were found in all comparisons: Coast and Inland regions (*F*_ST_ = 0.014; *p* = 0.20); Coast and Center (*F*_ST_ = −0.018; *p* = 0.91); and Center and Inland (*F*_ST_ = 0.021; *p* = 0.11).

The native lineages A2, B2, and C1 found in this study were compared with those from admixed populations in South America with published mitogenome data. Namely, 114 mitogenomes from Ecuador (Brandini et al. 2018), 16 from Chile (De Saint Pierre et al. 2012), 92 from Argentina (García et al. 2021), 84 from Paraguay (Simão et al. 2019), 118 from Colombia, and 82 from Peru (1000 Genomes Project Consortium et al. 2015) were included. The haplotypes from this study are well differentiated from those previously reported in South American populations (Figures S1–S4), except for one sample from haplogroup C1, whose haplotype was described in another sample from Colombia. It is noteworthy that no shared haplotypes were observed among the samples in this study, except within haplogroup B2d. Haplogroup B2d was only described in the Colombian samples from the dataset. Nonetheless, the Andean samples from the 1000 genomes present haplotypes in separate branches from those of our Caribbean samples.

The African lineages L1c, L2a, L3d, and L3e found in this study were compared with those from South American and African populations with published mitogenome data. Namely, 9 mitogenomes from Colombia and 3 from Peru (1000 Genomes Project Consortium et al. 2015), 7 from Paraguay (Simão et al. 2019), 170 from Angola (Barbieri et al. 2014), 85 from Sierra Leone, 113 from Gambia, and 108 from Nigeria (1000 Genomes Project Consortium et al. 2015) were included. The results of this analysis show the absence of haplotype sharing between our samples and those from other regions of South America, as well as Africa (Figures S5–S8). Within the samples analyzed in this study, only two pairs from the L2al haplogroup share the same haplotype, with both pairs consisting of one sample from the Coast and another from the Center. Although no matches were found, some samples from the Caribbean Coast are close to those from Colombia and from some African populations, which will be discussed further below.

### 3.3 Y Chromosome

The Y‐Chr haplogroups obtained in the three regions are presented in Table 2.

**TABLE 2:** Y‐Chr haplogroup composition and frequencies in the Coast, Center, and Inland regions, with the proportions of haplogroups that can be assigned to an African (AFR), Native American (NAM), or European (EUR) origin.

Haplogroups	Coast	Center	Inland
AFR origin	36.4%	38.1%	15.0%
A1a‐M31	1	—	—
B2a1a1a1‐M109	—	1	—
E1a‐M132	1	1	1
E1b1a1a1a1c1‐CTS5038*(xM191, Z36721, Z6023)	1	1	—
E1b1a1a1a1c1a1a‐U174/P252*(xFT212537, Y96183)	1	3	—
E1b1a1a1a2a1‐U209*(xZ37284, U290)	2	—	—
E1b1a1a1a2a1a3b1a‐U290*(U181, L649)	2	—	—
E1b1b1a1a1‐V12*(xFTA77423, BY8405, Y133233, BY8198,V32)	—	—	1
E2b1a‐BY36751/M85*(xCTS5716)	—	1	1
R1b1b‐V88*(xV69)	—	1	—
NAM origin	0.0%	4.8%	5.0%
Q1b1a1a‐M3*(xM19, M194, P106, SA01, Z35616, BZ3401, Y18425)	—	1	1
EUR origin	63.6%	57.1%	80.0%
E1b1b1a1b1a‐V13/PF2211*(xE‐FGC44465, E‐Y58870)	—	1	—
G2a2b‐M406*(xL14, Z37368)	1	—	—
I1a2b‐Z138	—	—	1
I2a1a1a‐M26/L158	—	1	1
I2a1b1a2a1‐P78	—	1	—
J1a2a1a2‐P58/Y2919*(xFGC8196)	—	1	1
J1a2a1a2d2b2b2c‐YSC0000234*(xZ1884, Y5148, Y191714, Y81349, Y254730)	1	—	1
J1a2a1a2d2b2b2c4‐Z1884	—	1	—
J1a2b‐Z1828	—	1	—
J2a‐M410/Y12378	—	—	1
J2a1a1a2b2‐M67	—	1	—
Q2a1a‐L245*(xY18596, BZ314)	1	—	—
R1a1a1b2a1a2‐Y9	1	—	—
R1b1a1b‐M269*(xR‐L51, R‐Z2103)	—	—	1
R1b1a1b1a‐L51*(xL52)	—	—	1
R1b1a1b1a1a2‐P312	3	1	4
R1b1a1b1a1a2a1‐Z195	2	—	1
R1b1a1b1a1a2a1a1a1‐Z216*(xR‐Z214)	—	—	1
R1b1a1b1a1a2a1a1a1a‐Z214	4	2	1
R1b1a1b1a1a2a1b1a‐Z262	—	—	1
R1b1a1b1a1a2b1‐L2*(xR‐Z258, R‐Z49)	—	1	1
R1b1a1b1a1a2c1‐DF13	1	—	—
T1a1a1b2b2b‐L906	—	1	—

A total of 34 different haplogroups were detected. Most haplogroups were observed only once, especially those of European origin, being therefore more diverse. The three regions showed different ancestry profiles, with the European haplogroups being the most frequent ones.

The European pool comprises haplogroups frequently found throughout Europe, with the subclades within R1b1a1b‐M269 being the most represented in our sample (61%). This high frequency of R1b1a1b, with lower frequencies of lineages within macrohaplogroups G, I, J, and T, is similar to that observed in populations from Western Europe. A comparison was performed between the frequencies of haplogroups in the pool estimated to originate from Europe and those found in Portugal (Beleza et al. 2006), Iberia (Adams et al. 2008), France (Bekada et al. 2013), Italy (Boattini et al. 2013), Germany (Rębała et al. 2012), and Lebanon (Zalloua 2008). The samples were grouped, after reducing the resolution to match the data from other studies, into the following haplogroups: E1b1b1*‐M35, E1b1b1a1‐M78, E1b1b1b1‐M81, E1b1b1c‐M123, F(xGIJ)‐M213, G‐M201, I‐M170, J‐P209, K*‐M9, R‐M207, R1b1‐M269. The results obtained did not show statistically significant differences with the Iberian and French populations (−0.002 < *F*_ST_ < 0.005; *p* ≥ 0.19). However, the comparison with Italy, Germany, and Lebanon showed increasingly higher genetic distances (*F*_ST_ = 0.027, 0.076, and 0193, respectively), all of them statistically significant (*p* < 0.01). This result is consistent with Spanish influence during European colonization. It is worth noting the presence of Q2a1a‐L245, which is not common in Western European populations, although a Q‐M242 sample (not subtyped for downstream markers) was found in Portuguese Gypsies (Gusmão et al. 2008). This haplogroup can also be originated from other region of Europe, since it is found in Central and Eastern Europe in association with Jewish populations (Huang et al. 2018).

The African haplogroups were the second most represented in the three regions. Ten different African haplogroups were found in the Caribbean, inside the clades A, B, E, and R.

Both clades A and B are restricted to Africa. Haplogroup A1a‐M31 was found in a sample from the coast. The highest frequency of this lineage was described in Guinea‐Bissau (Rosa et al. 2007), not being common in other regions of the continent, which makes its origin from the Upper Guinea region more likely. The haplogroup B2a1a1a1, found in one sample from the Center, is distributed throughout Sub‐Saharan Africa in Bantu‐speaking populations (Cruciani et al. 2002; Beleza et al. 2005). According to Beleza et al. (2005), this haplogroup originated in the west and during the Bantu expansion, it was dispersed across Sub‐Saharan Africa toward the Southwest, Central, and Southeast regions. Its wide distribution makes it difficult to pinpoint the most likely region of origin.

Seventeen of the twenty samples from the African‐origin set were assigned to haplogroups within clade E, which is the most widespread throughout Africa. Within this clade, haplogroup E1b1a‐M2 (and its sub‐lineages) reaches frequencies above 80% in most Bantu populations of sub‐Saharan Africa (Beleza et al. 2005; De Filippo et al. 2011; Ansari‐Pour et al. 2012; Rowold et al. 2014). The wide distribution and lack of data from African populations with the same haplogroup resolution as this study prevents us from assigning a specific African origin to each of them. However, we can observe that the lineages found within E1b1a‐M2 are similar to those previously described in San Basillio de Palenque, an Afro‐descendant community founded by refugee slaves from Cartagena (Martínez et al. 2020). In that study, the possible provenance of these lineages was determined in a vast region of the western coast, from Angola to Bight of Benin.

A sample from the coast was classified as E1a‐M132, a haplogroup restricted to western Africa, with the highest frequencies reported in populations from Mali and Burkina Faso (Cruciani et al. 2002; De Filippo et al. 2011). The gradual decrease in its frequency toward the south makes its origin in the West African region, between the Bight of Benin and Upper Guinea, more likely.

Two samples belonging to haplogroup E2b1a‐M85 were also found in the Center and Inland regions. The highest frequencies of this haplogroup have been described in Nilo‐Saharan populations from East Africa, being rarer in populations on the west coast (Wood 2005; Gomes et al. 2010). Its geographical distribution, associated with historical data, suggests that Mozambique may have been its place of origin. Although we do not have data from this country for comparison, recent data show a frequency of 7% in Zimbabwe, its neighboring country (Nguidi et al. 2026).

The haplogroup R1b1b‐V88 was found in a sample from the Center. Except for rare sub‐lineages, R1b‐V88 is essentially restricted to the African continent, with high frequencies in populations of the central Sahel (D'Atanasio et al. 2018). In West Africa, this haplogroup was found in Gabon, Nigeria, and Equatorial Guinea (Berniell‐Lee et al. 2009; González et al. 2013; Nguidi et al. 2024), being absent from populations further south. Therefore, the region near the Bight of Biafra is the most likely starting point in Africa.

The Native American haplogroup Q1b1a1a‐M3 was found in just 5% of the samples in the Center and Inland regions and was absent in the Coast. This haplogroup is the most frequent in Native Americans and is widely spread all over South America (Huang et al. 2018). The lack of downstream mutations does not allow us to attribute it to any specific region of this sub‐continent (Köksal et al. 2022).

For the Y‐Chr, pairwise *F*_ST_ genetic distance was calculated based on the Y‐SNP haplogroup frequencies displayed in Table 2. Low genetic distances were found in all comparisons, not being statistically significant: Coast and Inland regions (*F*_ST_ = −0.005; *p* = 0.58); Coast and Center (*F*_ST_ = 0.001; *p* = 0.44); and Center and Inland (*F*_ST_ = −0.005; *p* = 0.57). In order to reduce possible biases caused by sample sizes, a new analysis was performed after grouping the data into the following macro‐haplogroups: A, B, E1a, E1b1, E2, Q1, Q2, G, I, J, R1a, R1b, and T. Supporting previously results, no statistically significant differences were found: Coast and Inland regions (*F*_ST_ = 0.017; *p* = 0.213); Coast and Center (*F*_ST_ = 0.013; *p* = 0.25); and Center and Inland (*F*_ST_ = 0.038; *p* = 0.10).

### 3.4 Analysis of Molecular Variance

Considering the results of *F*_ST_ pairwise comparisons, an Analysis of Molecular Variance (AMOVA) was performed to access population stratification by including (i) all populations in a single group and (ii) two groups: G1—Coast and Center, and G2—Inland region. AMOVAs were calculated using genotypic data from AIM‐SNPs and mtDNA sequences (haplotypes). The results from Grouping 1 (Table 3) show that most variation is within populations. The variation found between populations was statistically significant only for AIM‐SNPs. Grouping 2 revealed that the remaining variation is attributed to differences between G1 and G2, rather than within these groups, highlighting similarities between Coast and Center and differences with the Inland region. Nonetheless, the small sample size did not allow statistically significant differentiation to be detected for both the autosomal and mtDNA markers (Table 3).

**TABLE 3:** Results from the analysis of molecular variance (AMOVA) considering AIM‐SNP genotypes and mtDNA haplotypes, using different grouping strategies.

	% of variation			
	Among groups	Among populations within groups	Within populations	*p*
AIM‐SNPs				
Grouping 1	—	0.96	99.04	0.0019*
Grouping 2	1.27	0.14	98.59	0.3355**
mtDNA				
Grouping 1	—	1.42	98.58	0.1535*
Grouping 2	4.83	−1.74	96.91	0.3319**

*Note:* Grouping 1: the three regions in a single group; Grouping 2: Two groups, with Coast and Center versus Inland region; *among populations; **among groups.

### 3.5 Evaluation of Forensic Tools to Assess Biogeographic Ancestry and Pigmentation Traits

**FIGURE 4:**
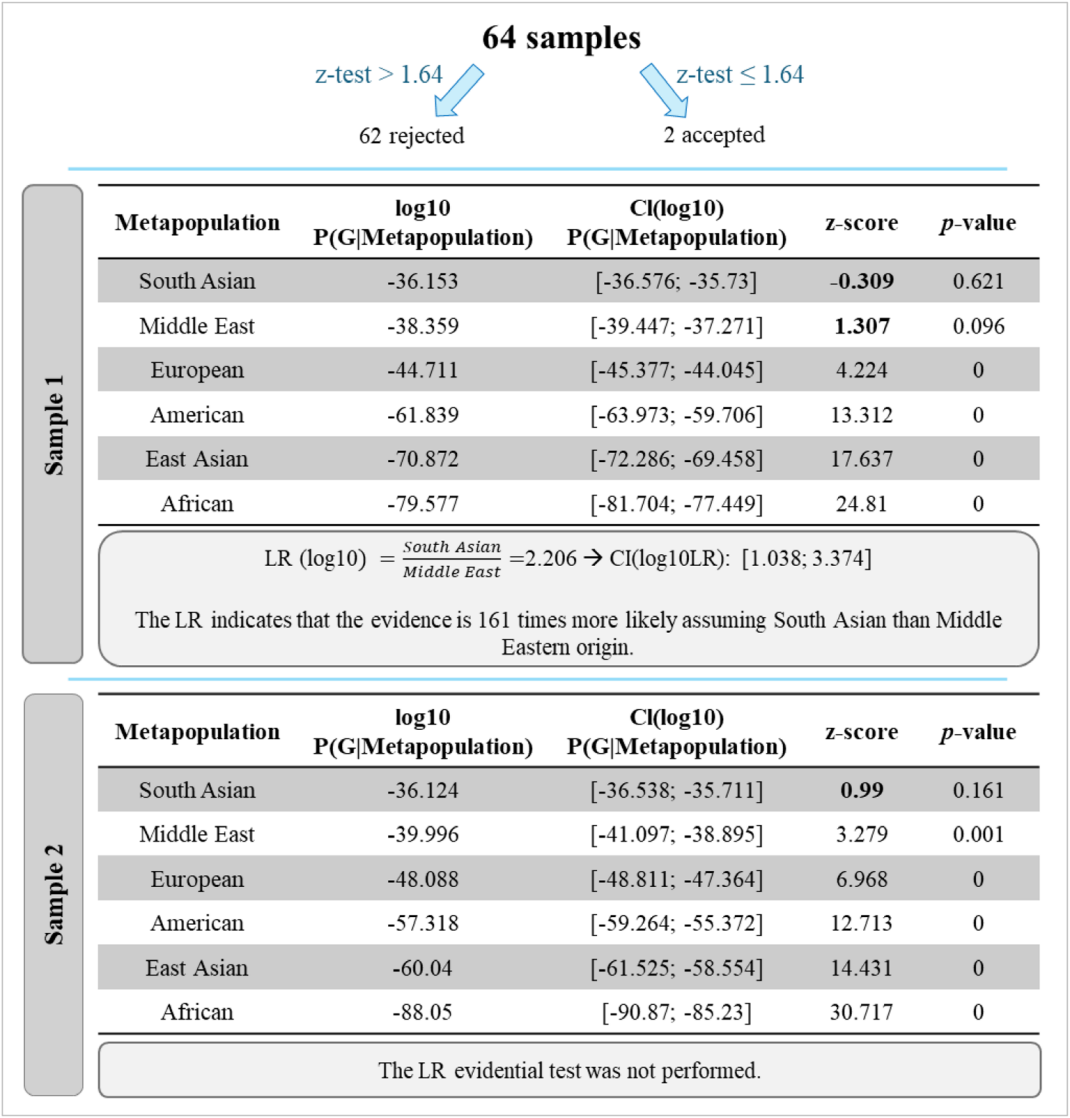
Flow chart of the results obtained with population assignments made by GenoGeographer. The values in bold indicate that the null hypothesis was accepted for the outlier test (*z‐*test ≤ 1.64).

The performance of the GenoGeographer (http://apps.math.aau.dk/aims/) in assigning admixed individuals from Colombia to their respective population was assessed. This tool was developed for forensic applications (Tvedebrink et al. 2017; Tvedebrink et al. 2018) and performs two types of analyses: (i) the outlier test, where the null hypothesis of “the AIM profile belongs to the reference population” can be accepted (*z‐*test ≤ 1.64) or rejected (*z‐*test > 1.64), and (ii) the Likelihood ratio (LR) evidential test, calculating the probability of the sample belonging to population A (*H1*) or population B (*H2*). The samples were tested using the six reference meta‐populations (Africa, Europe, America, East Asia, Middle East, and South Asia) with data available for the panel comprising the 115 AIM‐SNPs analyzed in this study (corresponding to the *VISAGE basic tool*; De la Puente et al. 2021). The hypothesis of belonging to any of the reference populations was rejected for most individuals (Figure 4). Two samples (3% of the cases) were accepted as potentially originating from at least one reference population. One sample was only accepted in the South Asian meta‐population, and since there was no alternative origin, the LR evidential test was not performed. The other sample was accepted in both South Asian and Middle Eastern meta‐populations. The LR evidential test showed that the genetic profile is about 160 times more likely when assuming South Asia than the Middle East as the population of origin.

**FIGURE 5:**
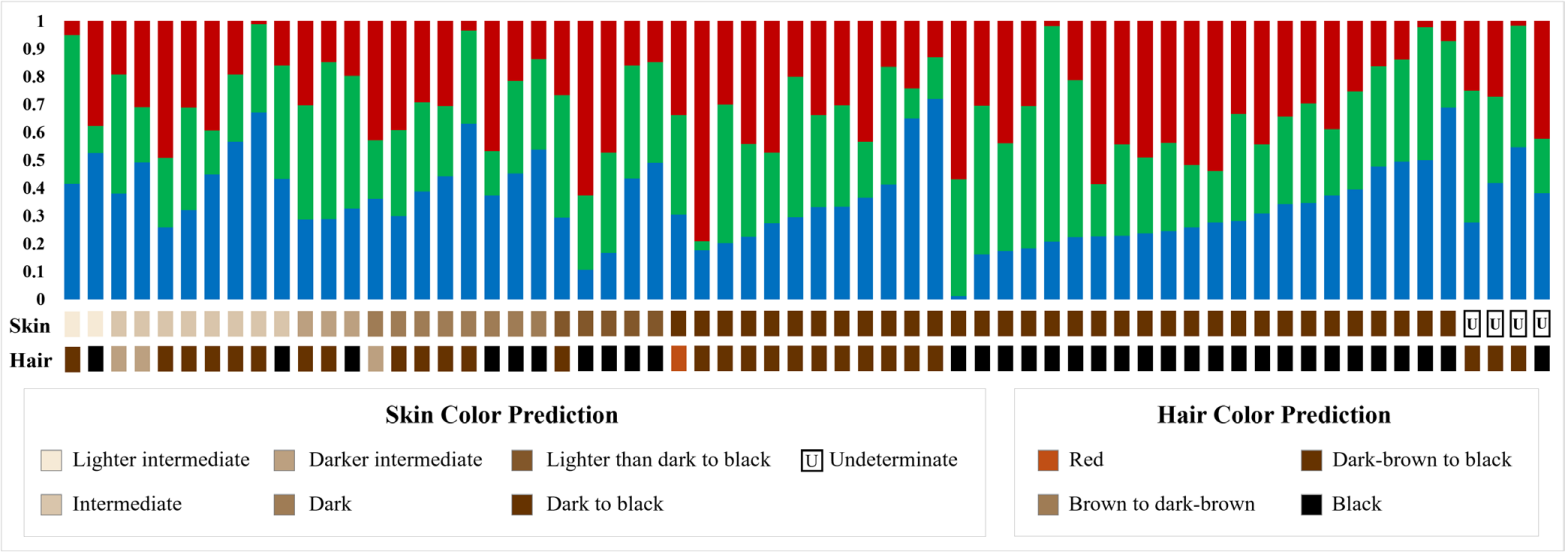
Results of hair and skin predictions correlated with corresponding European (EUR), African (AFR), and Native American ancestry proportions for each of the Caribbean samples included in this study.

The second forensic tool tested (HIrisPlex‐S1; https://hirisplex.erasmusmc.nl/) was developed for the determination of pigmentation‐related phenotypic traits based on 41 SNPs included in the COMBO panel. Significant dropouts were observed for the markers rs10756819 (BNC2 gene), rs1470608, and rs1545397 (OCA2 gene), all affecting skin color predictions. Most individuals were predicted to have brown eyes (*n* = 62), black to dark brown hair (*n* = 60), and skin tones ranging from intermediate to black (*n* = 60) (Figure 5). Only three samples had unusual predictions for the studied population, with blue eyes predicted for two individuals, and red hair for one individual. These less common phenotypes do not appear to correspond to recent migrants from Europe, as they correspond to individuals with relatively high proportions of non‐European ancestry. Namely, the red‐haired sample donor has 36% NAM and 34% AFR ancestries. For the blue‐eyed individuals, one has 15% NAM and 13% AFR ancestries, and the other has 33% NAM and 44% AFR.

When analyzed together with the ancestry results, variations in hair and skin color have a low correlation with the ancestry of the individuals, indicating that they most likely result from admixture events that occurred in colonial times, rather than recent migrations (Figure 5).

## 4 Discussion and Conclusions

In this study, the ancestry profiles of three regions within the Caribbean Department of Bolívar were investigated, at varying distances from Cartagena, a major entry port for Europeans and enslaved Africans arriving in South America during European colonization.

Regarding the autosomal ancestry profile, considering the entire population sample, a higher African ancestry was observed in Bolívar in comparison to that reported for other regions of the country, except the Pacific and Insular regions (Rojas et al. 2010; Ossa et al. 2016; Mogollón Olivares et al. 2020). Differences were found from those previously reported for the Caribbean region (Ossa et al. 2016; Mogollón Olivares et al. 2020), with a greater African and Native contribution found in this study. This difference could be explained by the restricted focus on the Department of Bolívar in this work, and not on the entire Caribbean region. The fact that the port of Cartagena is in the Department of Bolívar justifies the increase in African ancestry, at the expense of a decrease in European ancestry. This explanation is consistent with the difference in ancestry reported by Rojas et al. (2010) between Bolívar and Magdalena, another department in the Caribbean region.

As for mtDNA, in comparison to that reported for Andean populations and other Colombian regions (Rojas et al. 2010; Castillo et al. 2023; Castillo et al. 2025), a higher proportion of African lineages was observed accompanied by a decrease in Native American lineages, except for the Pacific. Maternal lineages of Eurasian origin were not detected in this study, although they have been reported among populations from other country regions at low frequencies (usually below 6%).

Regarding the Y chromosome, the prevalence of paternal lineages of European origin are in accordance with results previously observed in admixed Colombian populations (Rojas et al. 2010). However, the frequency of African lineages is higher than that found in most Andean populations, and the frequency of Native American lineages is lower. In fact, only 5% of the lineages found in this study are of Native American origin, similar to results reported in another study in populations from the Department of Bolívar (Noguera et al. 2014).

In general, the integrated analysis of uniparental and biparental transmission markers reveals a genetic profile of the three regions analyzed that reflects the historical processes that occurred in the Caribbean. Coast and Center populations show a higher overall African ancestry, justified by their proximity to the port areas where most Europeans and enslaved Africans settled during the colonial period. The region furthest from the coast preserves a greater Native American ancestry, observed in maternal and biparental inheritance, consistent with the displacement of indigenous groups to remote areas, promoted by the arrival of Europeans. However, Inland presents the highest proportion of European paternal lineages among the three regions, revealing a mating bias between European men and native women that almost replaced the native gene pool. In fact, in all regions studied, uniparental markers evidence sex‐biased mating events historically associated with South American populations.

It is worth highlighting that ancestry markers were used in this study strictly for the purpose of exploring the population history and patterns of migration, and the results obtained cannot be used to support any racial classifications or ethnic inferences. In fact, the research design does not involve community engagement and therefore reflects a general‐population sampling approach rather than community‐based research. The samples come from the general population and only information about the place of birth is available, preventing the use of the generated data to link genetics to social categories.

### 4.1 Comparison of the Overall Ancestry Profile of the Three Regions

**FIGURE 6:**
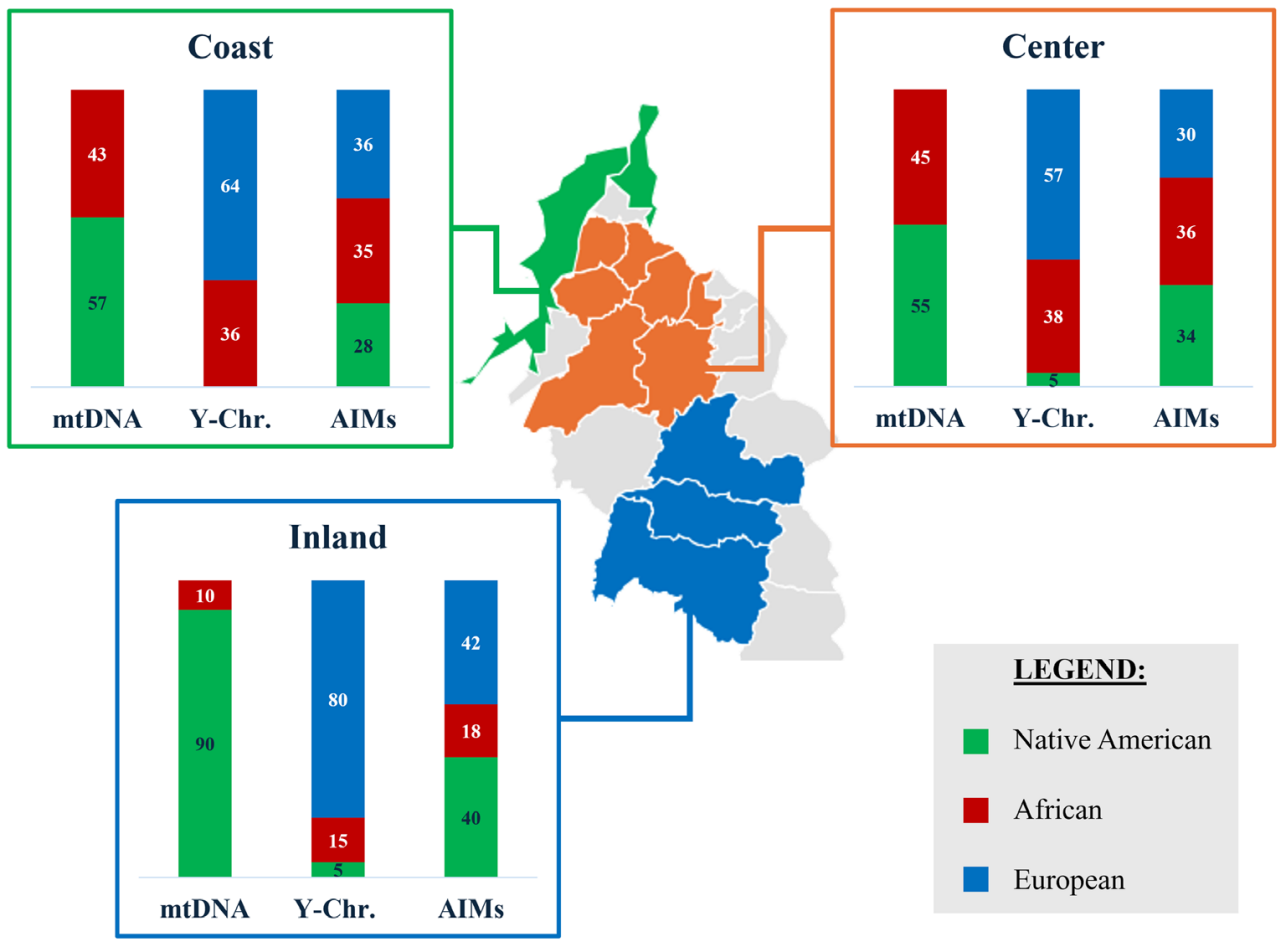
Continental ancestry proportions based on mtDNA, Y‐Chr, and AIMs in the three regions of Caribbean Colombia.

Continental admixture patterns for maternal, paternal, and biparental inheritance markers vary among the three regions studied (Figure 6).

The overall results show a similar profile between the Coast and Center regions for both autosomal and mtDNA markers, with just a slight increase of Native American Y‐Chr haplogroups in the Center. Genetic differentiation tests indicate no significant genetic distances between these two regions, concerning maternal, paternal, and biparental backgrounds. It is important to highlight that while these two regions exhibit similar average ancestries, the Coast region demonstrates greater intrapopulation variation (evidenced in STRUCTURE results and PCA; Figures 2 and 3, respectively), suggesting a higher degree of population stratification compared to the Center region.

The Inland region, on the other hand, shows an increase in Native ancestry for both autosomal markers and mtDNA, when compared to the Coast and Center regions. The frequency of paternal lineages of Native American origin remains low (not varying compared to the Center region), with European patrilineages showing the highest proportion in this region. It is worth noting that in the study by Noguera et al. (2014), a significantly higher frequency of Native American paternal lineages was found in a population from Bolívar, located in a region even further from the coast (Pinillos). African ancestry displays the lowest values in the Inland region for the three types of markers. Genetic differentiation tests indicate significant genetic distances among markers between the Inland region compared to the other two regions, except for Y‐Chr.

The increase in Native ancestry toward the interior of the territory is in accordance with the expected based on historical data, with records indicating the escaping of indigenous populations toward the interior of the country during European colonization. The decrease in African ancestry toward the interior shows the influence of Africans staying near the ports of arrival, where they were held for forced labor. The liberation movements of slaves and the creation of *palenques* far from the coast, although they may have contributed to a dispersion of African genetic heritage into the interior of the country, were not enough to homogenize the African contribution throughout the three regions studied.

### 4.2 Contrasting Maternal and Paternal Ancestries

To infer gender‐specific population dynamics and mating patterns, maternal and paternal lineage results were compared across the three regions.

In the coastal population sample, European maternal lineages were absent, which is consistent with the almost exclusive arrival of European men during colonial times, who replaced Native men, whose lineages were not detected in the sample from this region (Figure 6). The African maternal and paternal lineages, on the other hand, showed similar proportions, highlighting the presence of both men and women among the enslaved Africans who arrived on the coast. The Center region shows a pattern very similar to that of the Coast, likely explained by the geographical proximity and high mobility between these two regions. The maternal and paternal continental ancestry patterns observed in these two regions are concordant with sex‐biased mating. As frequently described in other South American populations, a strong mating bias is observed between European men and Native women. The genetic data alone, however, cannot confirm biased mating between European men and African women, as frequently seen in other South American admixed populations. In fact, there are two alternatives that explain the results: (i) the absence of a significant sex bias between European men and African women; or (ii) a sex bias not only between European men and African women, but also between African men and Native women.

In the interior region, there was a high preservation of Native maternal lineages and a sharp increase in paternal lineages of European origin. Once again, this pattern demonstrates the strong mating bias between European men and Native women. The higher proportion of paternal African lineages relative to maternal ones not only demonstrates that the liberation movements of enslaved people toward the interior of Bolívar involved a greater number of men but also implies the occurrence of sex‐biased matings between African men and Native women.

In all regions, the almost complete absence of paternal lineages of Native American origin is striking, highlighting the history of extinction faced by Native men and the replacement of the original paternal gene pool with European and African ones. In contrast, the preservation of the Native maternal component reflects the preferential assimilation of women from Native communities throughout the Bolívar territory.

### 4.3 Seeking Affinities Between Native American mtDNA Haplogroups in South America

To obtain a broader overview of the genetic similarities between the mitogenomes in the Caribbean sample and those from other South American populations, phylogenetic analyses were performed for the most frequent haplogroups in our sample.

The most common Native American lineages were A2 + (64) and B2d. The haplogroup A has a broad distribution throughout the continent, reaching the highest frequencies in the Caribbean coast of Venezuela and Colombia (Castro de Guerra et al. 2012; Yunis and Yunis 2013), which is further supported by our results. The phylogenetic analysis showed that most of our samples are placed in new branches separated by a high number of mutations from those previously described, with just two samples from haplogroup A2al being close (four mutational steps apart) to another one from Colombia (Figure S1). Furthermore, despite the differences between the haplotypes in this study and those of other populations, some sharing is observed at the level of the main A2 branches only with haplotypes from Peru.

The macro haplogroup B has a broad distribution in the Andean region, representing about 50% of the haplogroups in Peru, Northern Argentina, and Bolivia (Bobillo et al. 2010; Cardoso et al. 2013; Sandoval et al. 2016; 1000 Genomes Project Consortium et al. 2015). In Colombia, this haplogroup has frequencies between 40% and 50% in the Andean and Caribbean regions (Yunis and Yunis 2013). In the phylogenetic tree of B2b haplotypes (Figure S2), the three Caribbean samples were located on distinct branches from each other and from other South American samples. The closest haplotypes, in parallel branches, showed differences of 10 and 11 mutational steps with one sample from Peru and another from Colombia, respectively. In contrast, the haplogroup B2d is only represented in the Colombian samples (Figure S3), which is consistent with its main presence in North Colombia and the West region of Venezuela (EMPOP V4R14; consulted on 07/02/2026). All Caribbean B2d mitotypes clustered in a unique branch, separated from those comprising the Andean samples from the 1000 Genome Project.

Haplogroup C is widely spread in admixed populations from South America, being frequent in Brazil and Argentina (Bobillo et al. 2010; Dos Reis et al. 2019). Three of the samples in our dataset belong to haplogroup C1c3, and are close to another sample from Colombia (Figure S4). Genetic similarities between Caribbean and Andean samples from Colombia are also reinforced by a shared haplotype between these two population datasets.

A rare Native American lineage from haplogroup D1 was found in our dataset. This haplogroup is commonly found toward southern South America, reaching 40% frequency in Argentina and Chile (Bobillo et al. 2010; Gómez‐Carballa et al. 2016).

In summary, the vast majority of Native American haplotypes found in the Caribbean were new, not grouping with those already described in other regions of South America. The results of these analyses highlight the high variability of Native American lineages that are yet to be described, which makes it difficult to draw definitive conclusions about relationships between populations. However, we can observe that some haplotypes found in the Caribbean were close to others described in samples from the Colombian Andes (1000 Genomes Project Consortium et al. 2015), supporting gene flow between these two regions. Only one sample of haplogroup D1 allows us to infer a link to the Southern Cone, although this likely resulted from recent migration.

### 4.4 Searching for the African Origin of the Maternal Lineages

The arrival of enslaved Africans modified the gene pool of South American populations, with a differential impact across the subcontinent. Given the importance of understanding the African roots of populations with high African ancestry, such as those in the Caribbean, a phylogenetic analysis encompassing African haplogroups was conducted. To infer the origins of the most representative African lineages in Caribbean Colombia, a phylogenetic analysis was performed considering South American and African populations.

The most frequent haplogroup in our sample was L2a1, which is broadly distributed in the continent and reaches the highest frequencies in Central Africa (Pereira et al. 2001; Salas et al. 2002; Rosa and Brehem 2011). Although haplotypes identical to those in our sample were not found in African populations, the phylogenetic tree of L2a1 shows proximity to haplotypes found in Angola and Nigeria (Figure S6). Namely, the Caribbean samples classified within L2a1a2 and L2a1c4a cluster with others from these two African regions. A sample from L2a1c5 is close to one from Nigeria, and two shared haplotypes from Caribe and one from Nigeria are positioned on parallel branches sharing the variants 5147 and 16,309.

The L1c and L3e lineages present different distributions in Africa (Rosa and Brehem 2011). L1c reaches the highest frequencies in Central Africa, and is also common in Bantu groups from Angola, with frequencies between 18% and 25% (Beleza et al. 2005; Coelho et al. 2009). Meanwhile, L3e is widely frequent throughout the territory, comprising around one‐third of the L3 lineages in Sub‐Saharan Africa (Coelho et al. 2009). These lineages were found at a low frequency in our population, both represented by two samples. In the L1c phylogenetic tree (Figure S5), we can observe that the sub‐haplogroup L1c3a1b is represented by two Colombian samples from the Caribbean and the Andes, and is not represented in the mitogenomes described for African populations. The Caribbean sample classified within L1c2, although distant from the others, is positioned in a region of the tree with a high frequency of haplotypes from Angola. Also close to Angola is an L3d1a1a sample (Figure S7). The other Caribbean sample, within the L3d1b1 haplogroup, is similar to others from Ghana and Sierra Leone, indicating a possible West African origin.

Haplogroup L3d is mainly distributed in West Sub‐Saharan Africa, reaching frequencies of 10% in the region (González et al. 2006). Despite the absence of shared haplotypes with samples from Africa, the three Caribbean samples form branches parallel to those of samples from Angola (Figure S8). While two of them, classified within L3e1d1 and L3e1e1, respectively, point to an origin in Southwest Africa, the origin of the other one is not so clear, as it is located in a branch within L3e2 that includes African samples with wide distribution.

In general, phylogenetic analyses highlighted the high diversity of African lineages in the Caribbean, most of which are not represented in the available data for African populations. This gap in the genetic characterization of African populations prevented us from inferring the origin of most of the lineages we observed in our sample. However, for some of them, it was possible to point to certain regions of Africa as the most likely origin. Our results are thus compatible with an African maternal contribution with an origin extending from the Upper Guinea in the West region to further south, encompassing the Loango and Angola regions in the Southwest. This result is consistent with historical records, highlighting the high diversity of origin of the slaves who arrived in Cartagena, mainly from the west coast of Africa, from Senegambia to Angola (Borucki et al. 2020; https://www.slavevoyages.org/). Although slaves brought from Mozambique also arrived in Cartagena at a later stage and on a smaller scale, due to the absence of mitogenomes of populations from southeastern Africa, it was not possible to establish links with that region.

Regarding African lineages from other South American countries, it is worth highlighting their low representation in the available mitogenome dataset, making inferences about their origins and relationships with the Caribbean difficult. The only relationship was observed with two samples (L1c3a1b and L2a1c4a) from Colombia, as expected since it was to the port of Cartagena that most of the enslaved people who were later taken to the Andean region arrived.

### 4.5 Evaluation of Forensic Tools for Assessing Biogeographic Ancestry and Pigmentation Traits

To assess their applicability to the admixed Caribbean population, two programs commonly used in forensic genetics were used to predict the population of origin and pigmentation phenotypes of the samples from this study, based on markers included in COMBO.

AIMs have been used in Forensic Science to infer the biogeographical origin of an unknown donor of biological evidence at a crime scene, to aid investigations when there are no suspects. However, the tools used for this purpose can present limitations and biases that must be considered. These tools aim to determine the likelihood ratios between alternative hypotheses regarding the population of origin. In fact, the inference is not related to determining a specific continental ancestry (differently from software used in population genetics), but rather to the probability of finding a particular genotypic combination in a certain population. This implies knowing the profile of the relevant population, that is, that the true population of origin is represented in the available databases. If this is not the case, the attribution may be made to a nearby population that is not actually of true origin. Certain populations also show potential bias related to recent and non‐homogeneous admixture profiles. In fact, our results show how complex it is to perform this type of inference in admixed populations due to the high variation and associated substructure.

The use of the GenoGeographer software revealed its limitations in predicting the continental origin of the Bolívar samples. In most cases, the program indicated the absence of a reference population compatible with our sample profiles. In only two instances, the software indicates a possible correspondence with populations from the Middle East and South Asia, which constitutes an incorrect attribution to the actual origin of these individuals. These results are not unexpected, as our population is not represented in any of the reference populations consulted. In fact, for the markers analyzed, there is reference data from America, but it includes populations with different ancestry profiles than that of our population. Studies indicate that admixture patterns in South American populations vary significantly between countries and regions within the same country, making it challenging to create a single reference population for all of them. This challenge in determining the biogeographic ancestry of individuals from some populations has been previously noted (Pfaffelhuber et al. 2020; Mogensen et al. 2020; Köksal et al. 2023; Salvo et al. 2024). In the absence of a reference for the population of origin, individuals from North and South American populations exhibited the highest rate of non‐attribution, with values above 70% (Mogensen et al. 2022). Studies on admixed American populations also showed that, even when the reference population is included, error rates are high in BGA inferences, using GenoGeographer or other similar software (Pfaffelhuber et al. 2020; Mogensen et al. 2020; Köksal et al. 2023). The results reflect the impact of the lack of representation from South American populations in reference databases and demonstrate the difficulty of BGA inferences in admixed populations with high interindividual ancestry variation.

Concerning the use of HIrisPlex‐S1 for Externally Visible Traits predictions, the interpretation of the results is more challenging, due to the lack of information on sample donors' phenotypes. Nevertheless, considering the general characteristics of the population and the ancestry results obtained, it can be anticipated that eye, hair, and skin color phenotypes will predominantly range from intermediate to dark. The results obtained fall within the expected range of variation, with only two individuals predicted to have blue eyes and one individual with red hair. For the two profiles predicted to belong to blue‐eyed individuals, the software indicates donors with hair color between dark brown and black, and a dark black skin color. It is worth mentioning that the associated probabilities of blue‐eyed individuals were relatively low (*p* = 0.65 and *p* = 0.51, respectively) when compared with those of intermediate and brown (*p* ≥ 0.77, except for one sample with *p* = 0.55 for brown). In the investigation by Walsh et al. (2012), a threshold of *p* ≥ 0.5 was associated with low accuracy probabilities of a correct phenotype, especially for intermediate colorations, and could also result in “undefined” data. However, a non‐conclusive prediction for blue eyes of *p* ≤ 0.7 should be addressed, with a lower accuracy of the phenotype. Thus, in cases similar to the samples studied, where the *p* < 0.7, intermediate eye color is highly possible rather than blue‐eyed individuals (Walsh et al. 2012). For the sample predicted to correspond to a red‐haired individual, dark‐black skin color and brown eyes were predicted, with an ancestry proportion of 34% AFR, 30% EUR, and 36% NAM. Although less common, populations with significant African or Native American and European admixture may exhibit individuals with contrasting phenotypes between eye color and hair and skin tones. To determine to what extent the phenotypes could be explained by the proportions of African, Native, and European ancestry, the results from both analyses were contrasted. No correlation was identified between them. In conclusion, although the accuracy of HIrisPlex‐S1 in predicting visible traits could not be evaluated due to the lack of phenotypic data from the donors, the results obtained show a complexity of phenotypes that are difficult to predict based solely on the ancestry profile within the population studied.

## Author Contributions

**Masinda Nguidi:** methodology, writing – original draft, conceptualization, writing – review and editing, formal analysis, investigation, data curation, validation, visualization. **Christina Amory:** data curation, writing – review and editing, validation, methodology, investigation, formal analysis. **Catarina Xavier:** data curation, writing – review and editing, validation, formal analysis. **Gabriela Huber:** data curation, writing – review and editing, investigation. **Filipa Simão:** writing – review and editing, data curation, formal analysis. **Beatriz Martinez:** writing – review and editing, resources, investigation. **Luis Caraballo:** resources, writing – review and editing. **Leonor Gusmão:** conceptualization, formal analysis, supervision, funding acquisition, project administration, resources, writing – original draft, writing – review and editing, visualization. **Walther Parson:** supervision, writing – review and editing, project administration, resources, funding acquisition, methodology, data curation, investigation, validation.

## Funding

M.N. was financed by the Coordenação de Aperfeiçoamento de Pessoal de Nível Superior—Brasil (CAPES)—Finance Code 001. L.G. was supported by FAPERJ—Fundação Carlos Chagas Filho de Amparo à Pesquisa do Estado do Rio de Janeiro (proc. SEI‐260003/003492/2022) and by Conselho Nacional de Desenvolvimento Científico e Tecnológico (CNPq), Brazil (proc. ref. 315007/2023‐0).

## Conflicts of Interest

The authors declare no conflicts of interest.

## Supporting information


**Figure S1:** Phylogenetic trees with geographic origin and frequencies of macrohaplogroup A in Caribbean Colombia and South America. The countries are represented in different colors (detailed in the “Legend”). The mutated positions are detailed in each node. Mitogenomes haplotypes were used and the following Indel positions were discarded: 16193.xC, 309.xC, 315.xC, 523‐524del, 524.xC.
**Figure S2:** Phylogenetic trees with geographic origin and frequencies of haplogroup B2b in Caribbean Colombia and South America. The countries are represented in different colors (detailed in the “Legend”). The mutated positions are detailed in each node. Mitogenomes haplotypes were used and the following Indel positions were discarded: 16193.xC, 309.xC, 315.xC, 523‐524del, 524.xC.
**Figure S3:** Phylogenetic trees with geographic origin and frequencies of haplogroup B2d in Caribbean Colombia and South America. The countries are represented in different colors (detailed in the “Legend”). The mutated positions are detailed in each node. Mitogenomes haplotypes were used and the following Indel positions were discarded: 16193.xC, 309.xC, 315.xC, 523‐524del, 524.xC.
**Figure S4:** Phylogenetic trees with geographic origin and frequencies of haplogroup C1 in Caribbean Colombia and South America. The countries are represented in different colors (detailed in the “Legend”). The mutated positions are detailed in each node. Mitogenomes haplotypes were used and the following Indel positions were discarded: 16193.xC, 309.xC, 315.xC, 523‐524del, 524.xC.
**Figure S5:** Phylogenetic trees with geographic origin and frequencies of haplogroup L1c in Caribbean Colombia and Africa. The countries are represented in different colors (detailed in the “Legend”). The mutated positions are detailed in each node. Mitogenomes haplotypes were used and the following Indel positions were discarded: 16193.xC, 309.xC, 315.xC, 523‐524del, 524.xC.
**Figure S6:** Phylogenetic trees with geographic origin and frequencies of haplogroup L2a in Caribbean Colombia and Africa. The countries are represented in different colors (detailed in the “Legend”). The mutated positions are detailed in each node. Mitogenomes haplotypes were used and the following Indel positions were discarded: 16193.xC, 309.xC, 315.xC, 523‐524del, 524.xC.
**Figure S7:** Phylogenetic trees with geographic origin and frequencies of haplogroup L3d in Caribbean Colombia and Africa. The countries are represented in different colors (detailed in the “Legend”). The mutated positions are detailed in each node. Mitogenomes haplotypes were used and the following Indel positions were discarded: 16193.xC, 309.xC, 315.xC, 523‐524del, 524.xC.
**Figure S8:** Phylogenetic trees with geographic origin and frequencies of haplogroup L3e in Caribbean Colombia and Africa. The countries are represented in different colors (detailed in the “Legend”). The mutated positions are detailed in each node. Mitogenomes haplotypes were used and the following Indel positions were discarded: 16193.xC, 309.xC, 315.xC, 523‐524del, 524.xC.

**Table S1:** Ancestry proportions of each individual based on the 115 AIM‐SNP profiles, with samples from coast, center and inner regions of Bolívar.
**Table S2:**: List of mtDNA complete genome haplotypes and haplogroups in the population samples of coast, center and inner regions of Bolívar.

## Data Availability

All data generated in this work is available in supporting information and has been deposited in GenBank: accession numbers PX925172‐PX925234, for mitochondrial DNA sequences; Population ID: Caribbean Colombian, for the autosomal SNP at dbSNP.
