# Supplementary material for: Contrasting Maternal, Paternal, and Biparental Ancestry of Populations From the Caribbean Coast of Colombia
Source: Am J Biol Anthropol. 2026 Apr 9;189(4):e70242. doi: 10.1002/ajpa.70242 (PMC13062887; doi:10.1002/ajpa.70242)
Supplement: Supplementary file 1 — Figure S1: Phylogenetic trees with geographic origin and frequencies of macrohaplogroup A in Caribbean Colombia and South America. The countries are represented in different colors (detailed in the “Legend”). The mutated positions are detailed in each node. Mitogenomes haplotypes were used and the following Indel positions were discarded: 16193.xC, 309.xC, 315.xC, 523‐524del, 524.xC. Figure S2: Phylogenetic trees with geographic origin and frequencies of haplogroup B2b in Caribbean Colombia and South America. The countries are represented in different colors (detailed in the “Legend”). The mutated positions are detailed in each node. Mitogenomes haplotypes were used and the following Indel positions were discarded: 16193.xC, 309.xC, 315.xC, 523‐524del, 524.xC. Figure S3: Phylogenetic trees with geographic origin and frequencies of haplogroup B2d in Caribbean Colombia and South America. The countries are represented in different colors (detailed in the “Legend”). The mutated positions are detailed in each node. Mitogenomes haplotypes were used and the following Indel positions were discarded: 16193.xC, 309.xC, 315.xC, 523‐524del, 524.xC. Figure S4: Phylogenetic trees with geographic origin and frequencies of haplogroup C1 in Caribbean Colombia and South America. The countries are represented in different colors (detailed in the “Legend”). The mutated positions are detailed in each node. Mitogenomes haplotypes were used and the following Indel positions were discarded: 16193.xC, 309.xC, 315.xC, 523‐524del, 524.xC. Figure S5: Phylogenetic trees with geographic origin and frequencies of haplogroup L1c in Caribbean Colombia and Africa. The countries are represented in different colors (detailed in the “Legend”). The mutated positions are detailed in each node. Mitogenomes haplotypes were used and the following Indel positions were discarded: 16193.xC, 309.xC, 315.xC, 523‐524del, 524.xC. Figure S6: Phylogenetic trees with geographic origin and frequencies of haplogroup L2 [file AJPA-189-e70242-s002.pdf]

Supplementary Figures

Nguidi et al. (2026). Contrasting maternal, paternal and biparental ancestry of populations from the Caribbean coast of Colombia

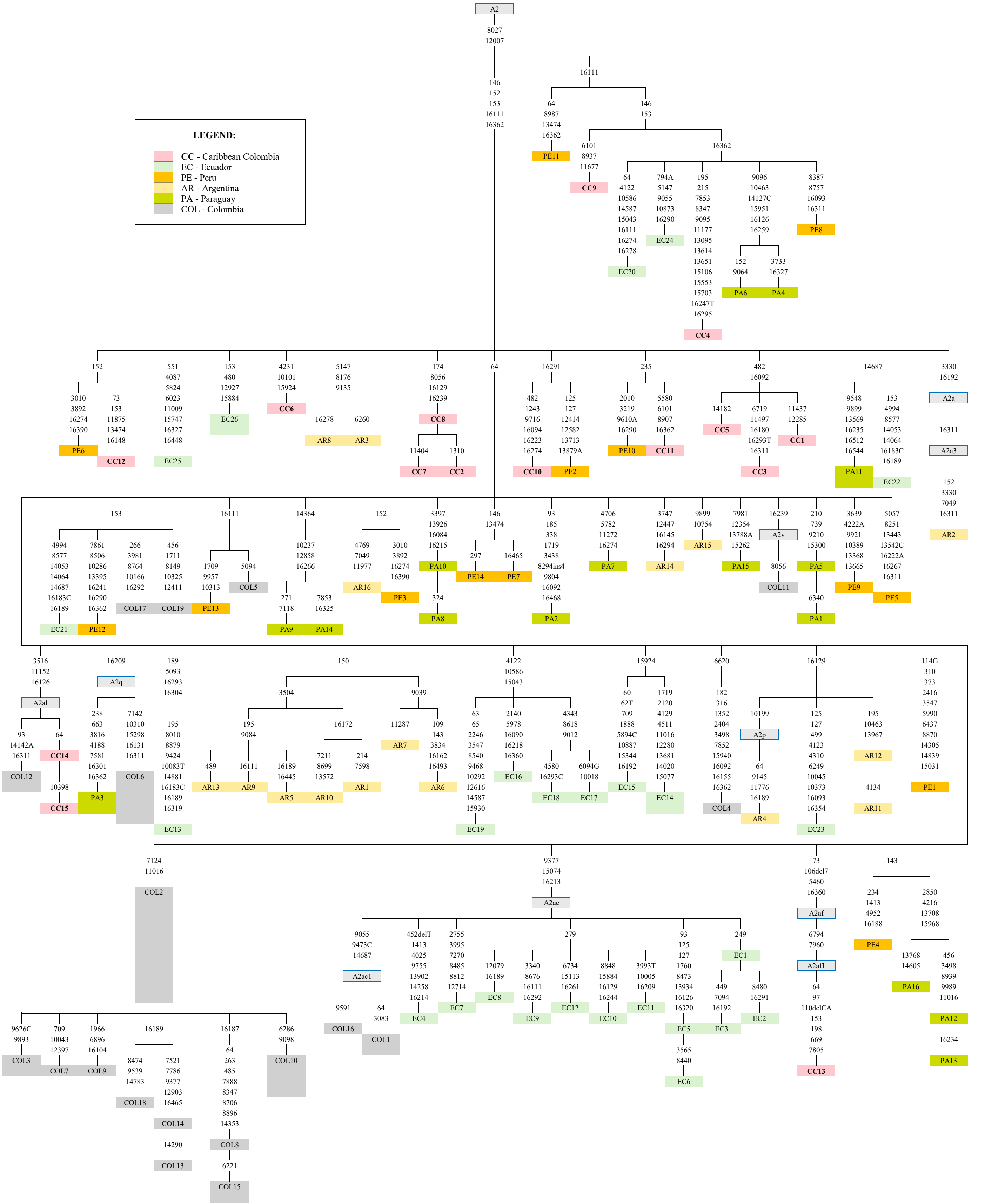

Supplementary Fig. S1 Phylogenetic trees with geographic origin and frequencies of macrohaplogroup A in Caribbean Colombia and South America. The countries are represented in different colors (detailed in the "Legend"). The mutated positions are detailed in each node. Mitogenomes haplotypes were used and the following Indel positions were discarded: 16193.xC, 309.xC, 315.xC, 523-524del, 524.xC.

References: EC: Brandini et al. *Molecular Biology and Evolution* (2018), doi:10.1093/molbev/msx267; PE and COL: 1000 Genomes Project Consortium et al. *Nature* (2015), doi:10.1038/nature15393; AR: García et al. *Human Molecular Genetics* (2021), doi:10.1093/hmg/ddab105; PA: Simão et al. *Forensic Science International: Genetics* (2019), doi:10.1016/j.fsigen.2018.12.007.

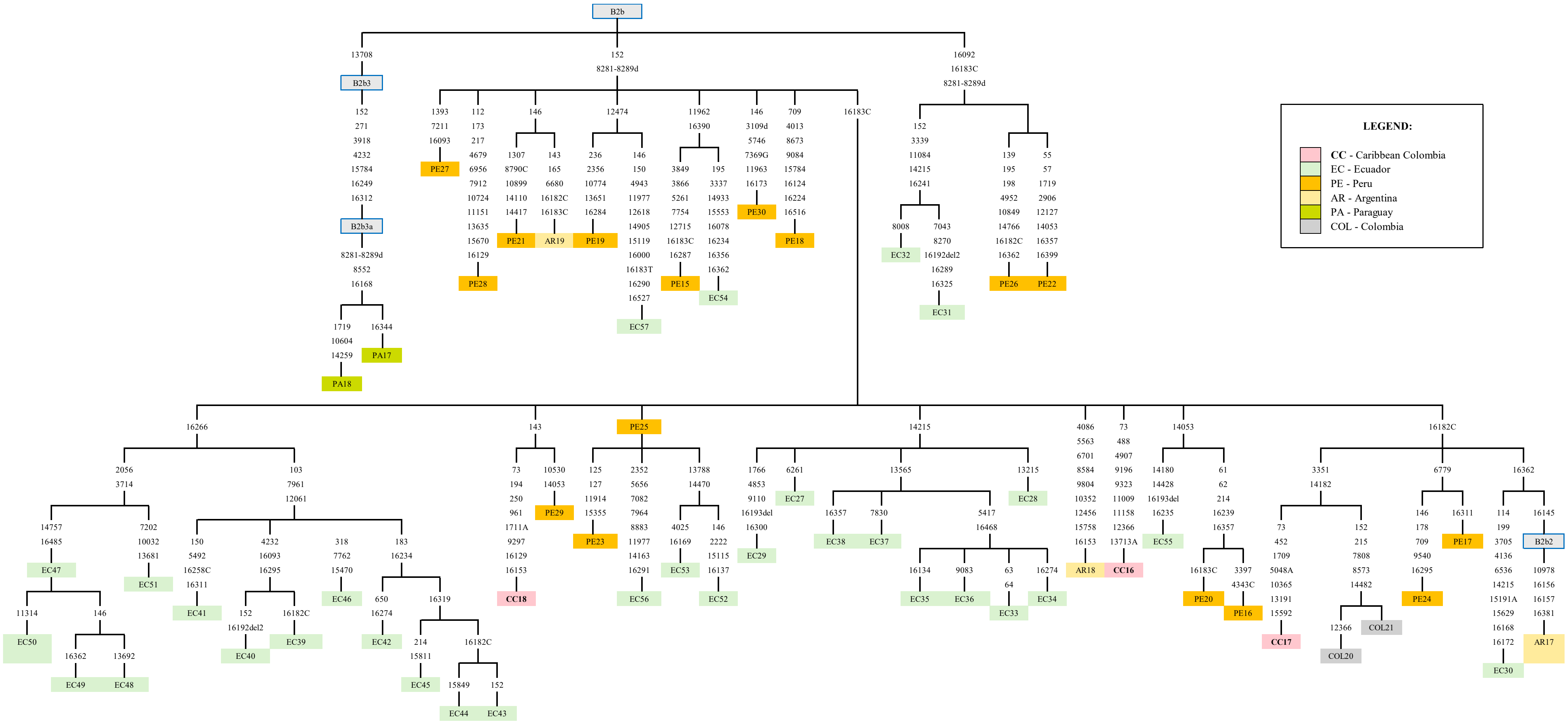

Supplementary Fig. S2 Phylogenetic trees with geographic origin and frequencies of haplogroup B2b in Caribbean Colombia and South America. The countries are represented in different colors (detailed in the "Legend"). The mutated positions are detailed in each node. Mitogenomes haplotypes were used and the following InDel positions were discarded: 16193.xC, 309.xC, 315.xC, 523-524del, 524.xC.

References: EC: Brandini et al. *Molecular Biology and Evolution* (2018), doi:10.1093/molbev/msx267; PE and COL: 1000 Genomes Project Consortium et al. *Nature* (2015), doi:10.1038/nature15393; AR: García et al. *Human Molecular Genetics* (2021), doi:10.1093/hmg/ddab105; PA: Simão et al. *Forensic Science International: Genetics* (2019), doi:10.1016/j.fsigen.2018.12.007.

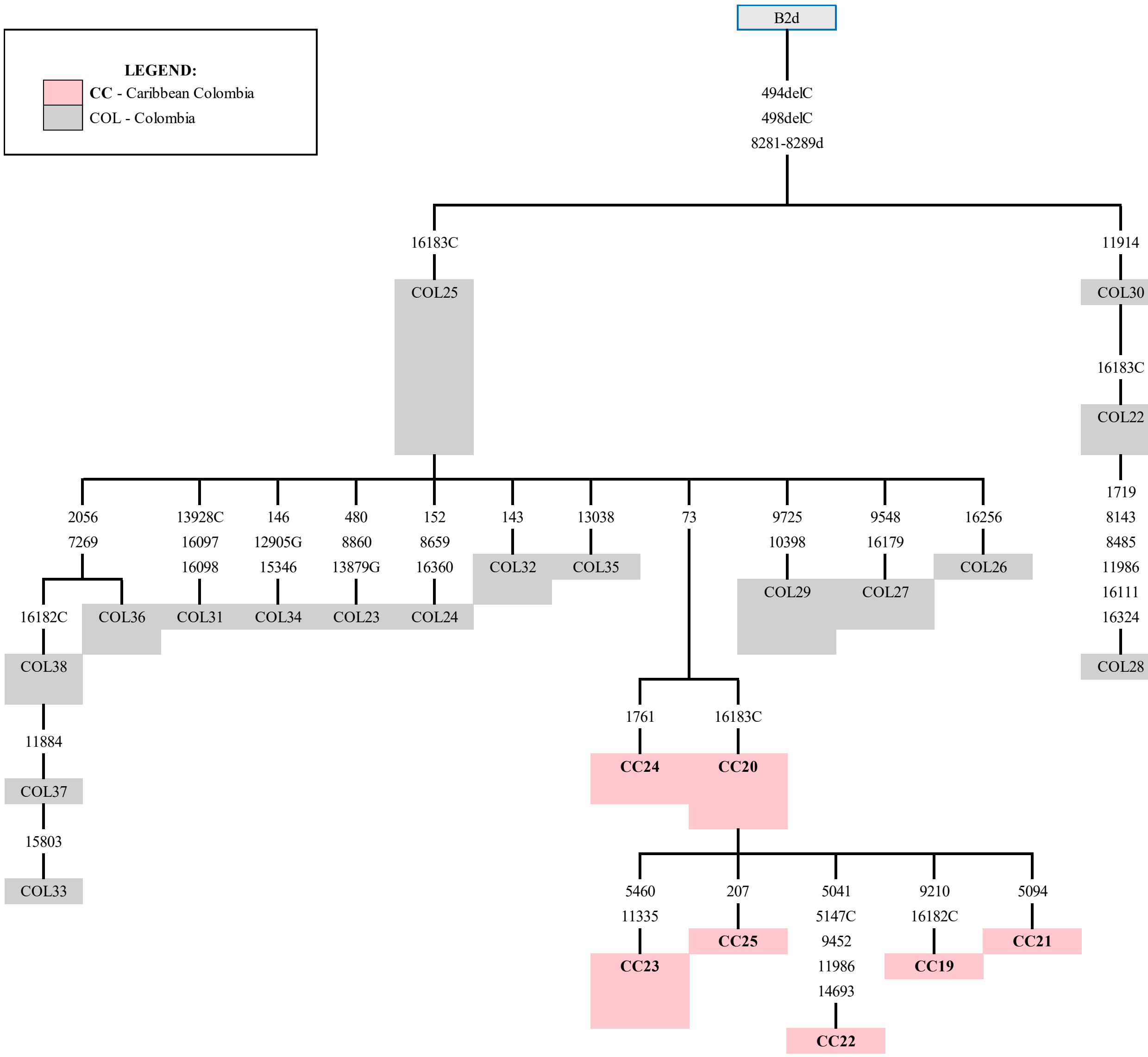

Supplementary Fig. S3 Phylogenetic trees with geographic origin and frequencies of haplogroup B2d in Caribbean Colombia and South America. The countries are represented in different colors (detailed in the "Legend"). The mutated positions are detailed in each node. Mitogenomes haplotypes were used and the following InDel positions were discarded: 16193.xC, 309.xC, 315.xC, 523-524del, 524.xC.

References: COL: 1000 Genomes Project Consortium et al. *Nature* (2015), doi:10.1038/nature15393.

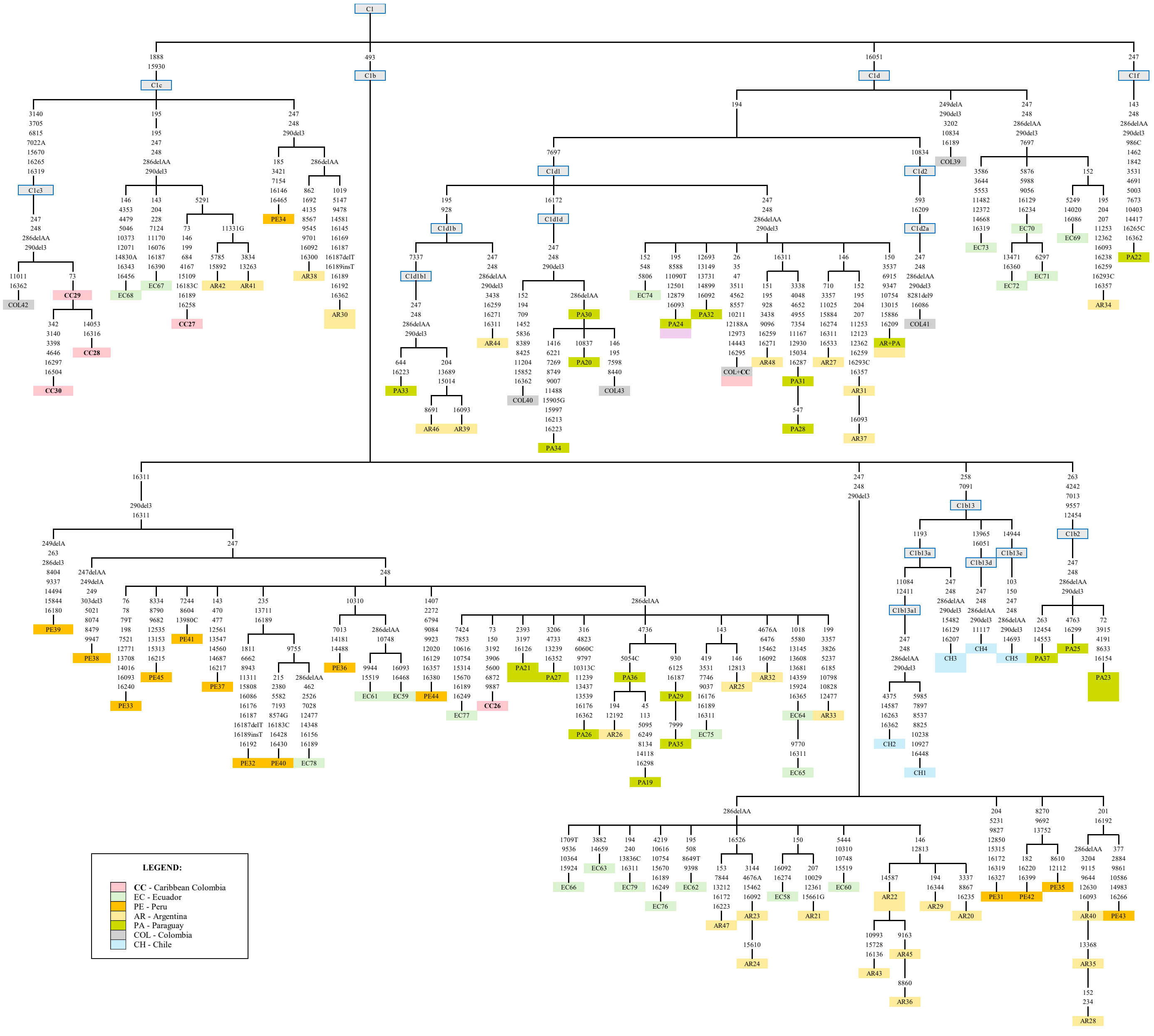

Supplementary Fig. S4 Phylogenetic trees with geographic origin and frequencies of haplogroup C1 in Caribbean Colombia and South America. The countries are represented in different colors (detailed in the "Legend"). The mutated positions are detailed in each node. Mitogenomes haplotypes were used and the following InDel positions were discarded: 16193.xC, 309.xC, 315.xC, 523-524del, 524.xC.

References: EC: Brandini et al. *Molecular Biology and Evolution* (2018), doi:10.1093/molbev/msx267; PE and COL: 1000 Genomes Project Consortium et al. *Nature* (2015), doi:10.1038/nature15393; AR: García et al. *Human Molecular Genetics* (2021), doi:10.1093/hmg/ddab105; PA: Simão et al. *Forensic Science International: Genetics* (2019), doi:10.1016/j.fsigen.2018.12.007; CH: de Saint Pierre et al. *PloS One* (2012), doi:10.1371/journal.pone.0051311.

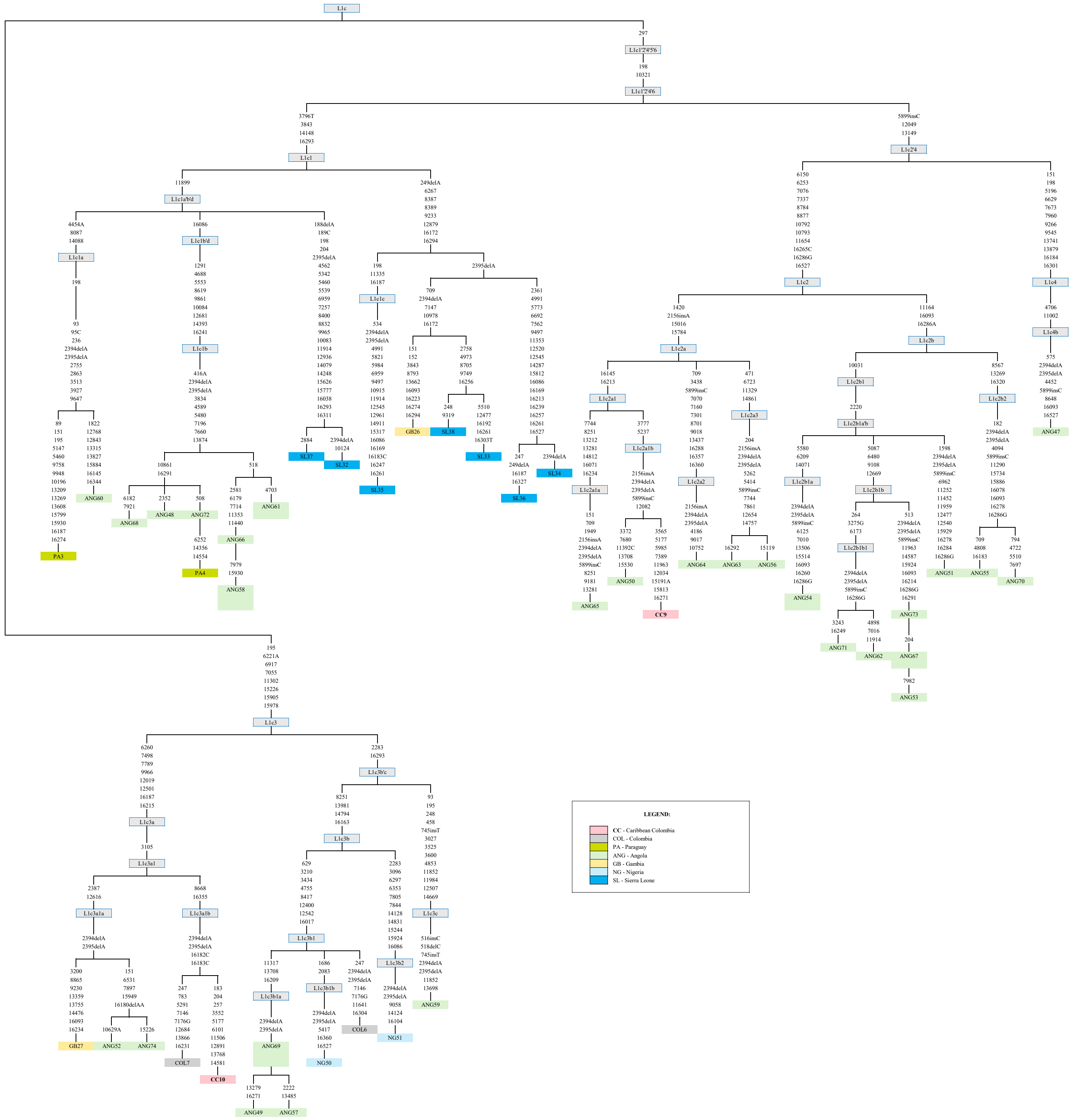

Supplementary Fig. S5 Phylogenetic trees with geographic origin and frequencies of haplogroup L1c in Caribbean Colombia and Africa. The countries are represented in different colors (detailed in the "Legend"). The mutated positions are detailed in each node. Mitogenomes haplotypes were used and the following InDel positions were discarded: 16193.xC, 309.xC, 315.xC, 523-524del, 524.xC.

References: COL, GB, NG, and SL: 1000 Genomes Project Consortium et al. *Nature* (2015), doi:10.1038/nature15393; PA: Simão et al. *Forensic Science International: Genetics* (2019), doi:10.1016/j.fsigen.2018.12.007; ANG: Barbieri et al. *PloS One* (2014). doi:10.1371/journal.pone.0099117.

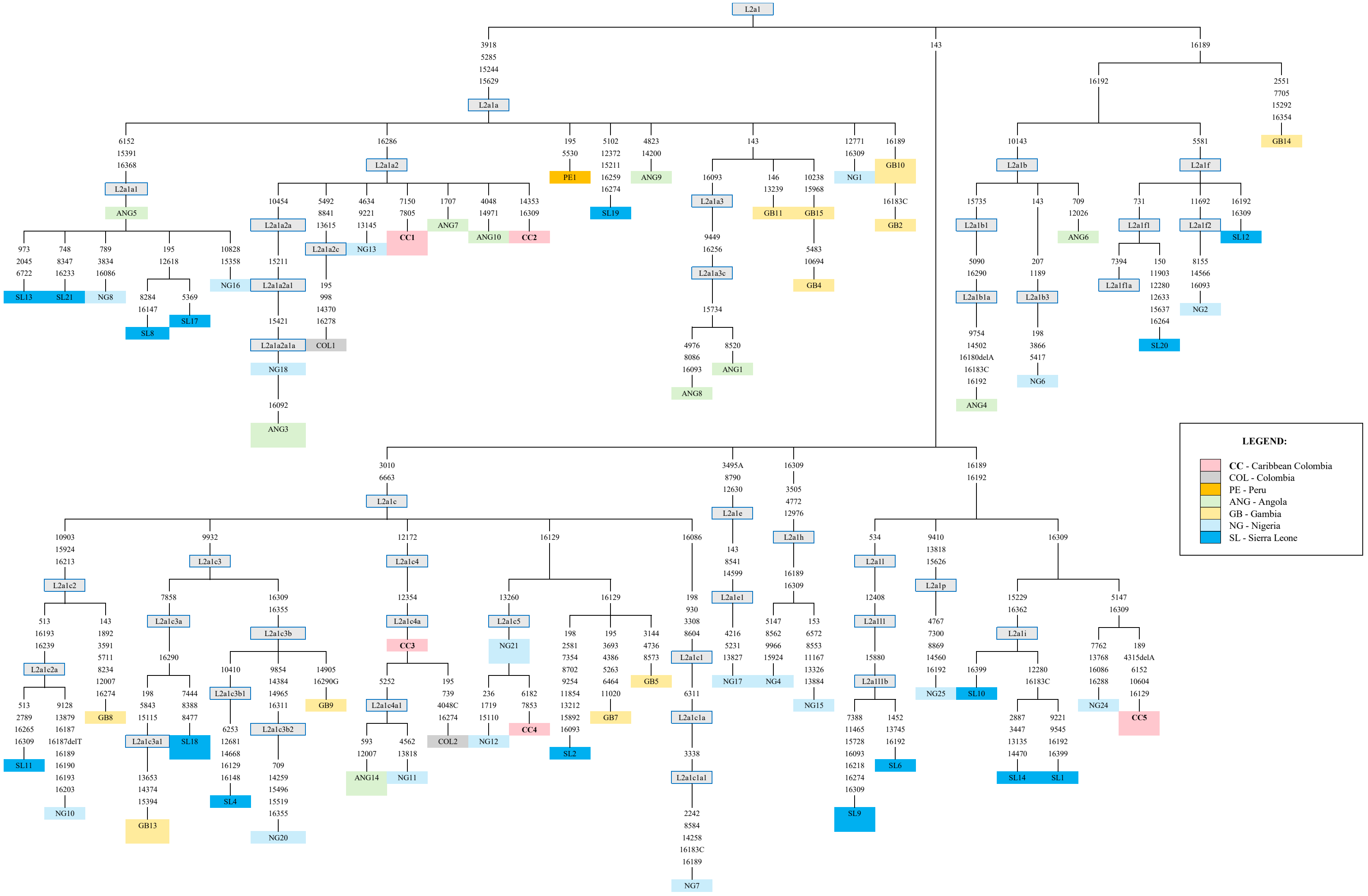

Supplementary Table S6 Phylogenetic trees with geographic origin and frequencies of haplogroup L2a in Caribbean Colombia and Africa. The countries are represented in different colors (detailed in the "Legend"). The mutated positions are detailed in each node. Mitogenomes haplotypes were used and the following InDel positions were discarded: 16193.xC, 309.xC, 315.xC, 523-524del, 524.xC.

References: <u>COL, PE, GB, NG, and SL</u>: 1000 Genomes Project Consortium et al. *Nature* (2015), doi:10.1038/nature15393; <u>PA</u>: Simão et al. *Forensic Science International: Genetics* (2019), doi:10.1016/j.fsigen.2018.12.007; <u>ANG</u>: Barbieri et al. *PloS One* (2014). doi:10.1371/journal.pone.0099117.

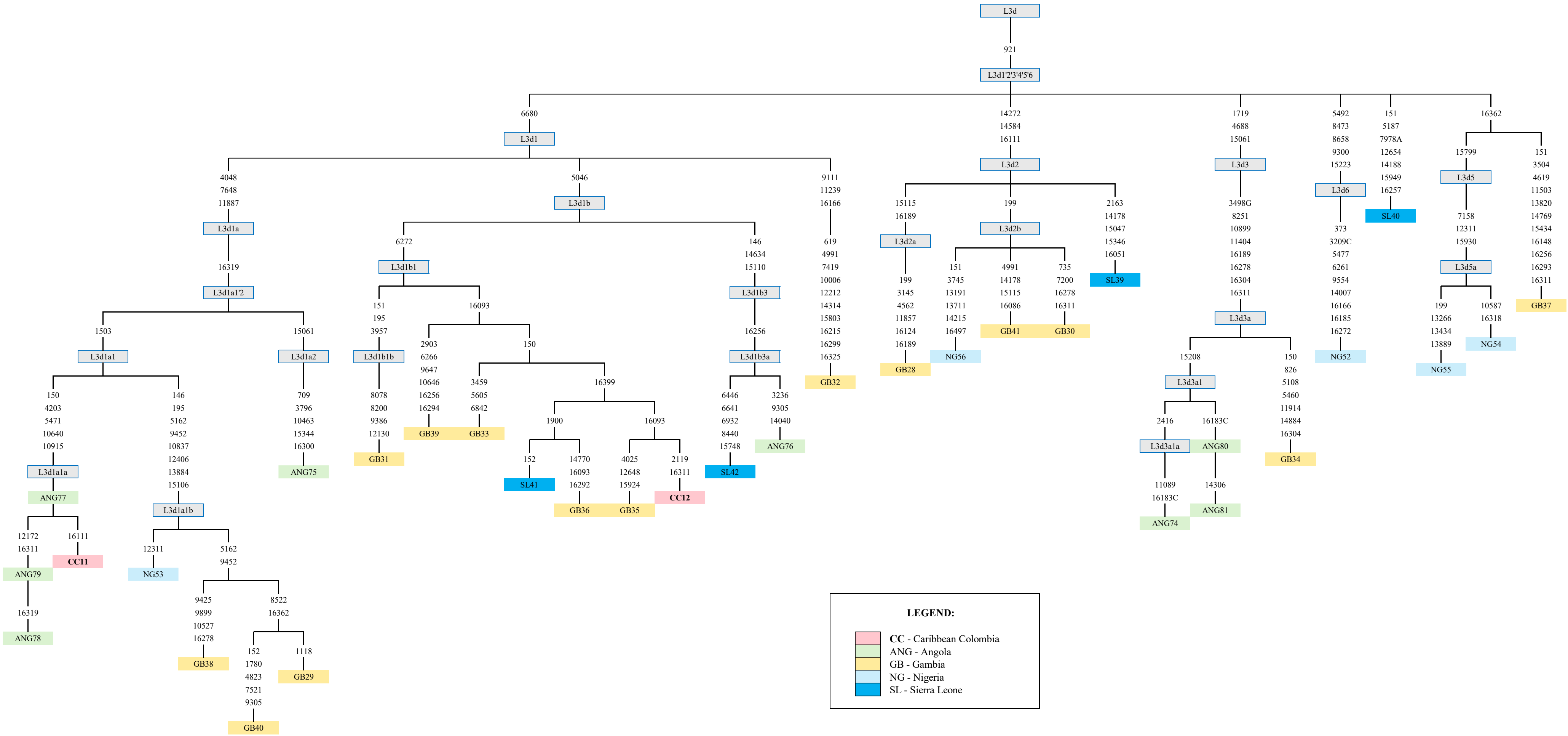

Supplementary Table S7 Phylogenetic trees with geographic origin and frequencies of haplogroup L3d in Caribbean Colombia and Africa. The countries are represented in different colors (detailed in the "Legend"). The mutated positions are detailed in each node. Mitogenomes haplotypes were used and the following InDel positions were discarded: 16193.xC, 309.xC, 315.xC, 523-524del, 524.xC.

References: ANG: Barbieri et al. *PloS One* (2014), doi:10.1371/journal.pone.0099117; GB, NG, and SL: 1000 Genomes Project Consortium et al. *Nature* (2015), doi:10.1038/nature15393

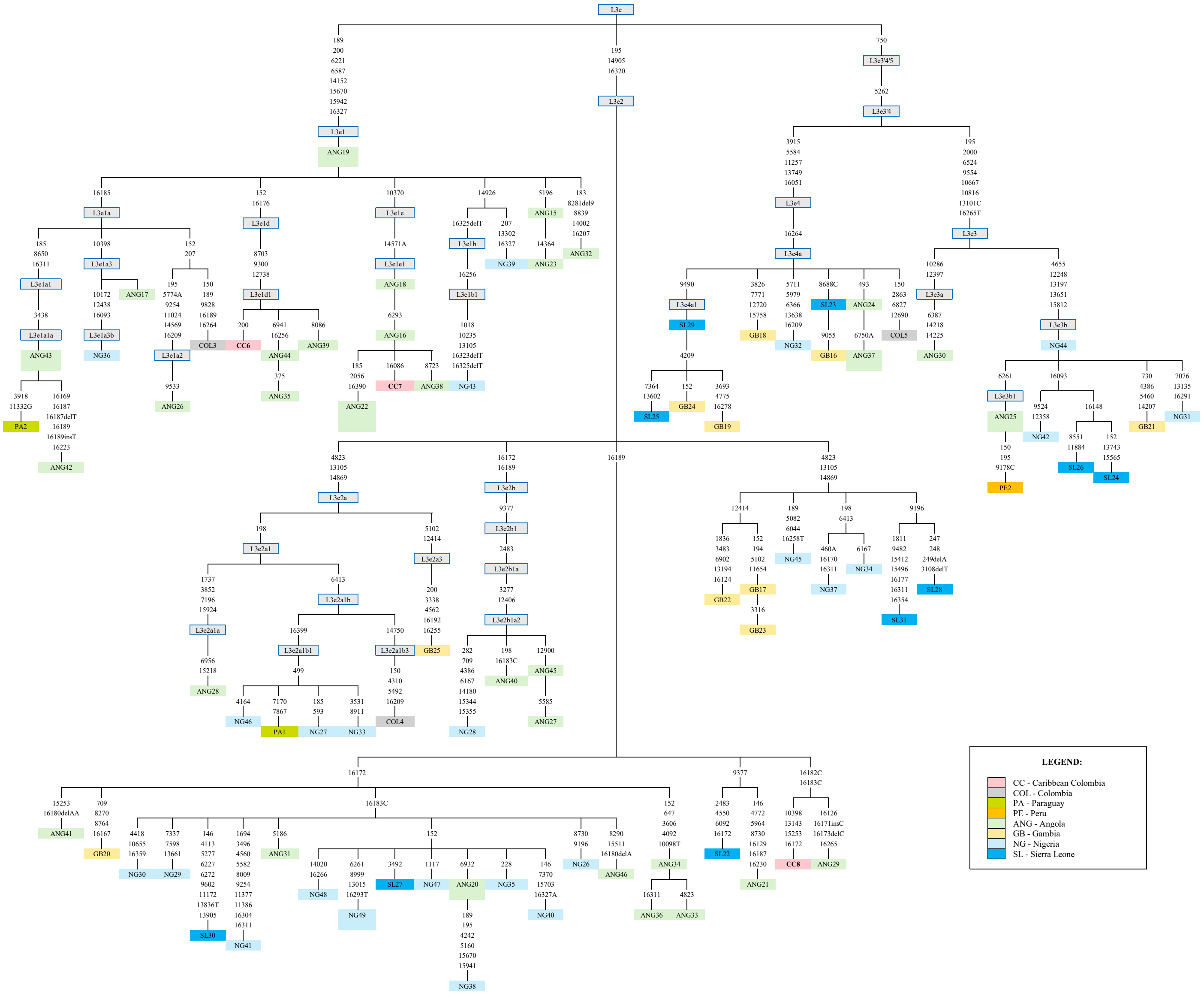

Supplementary Fig. S8 Phylogenetic trees with geographic origin and frequencies of haplogroup L3e in Caribbean Colombia and Africa. The countries are represented in different colors (detailed in the "Legend"). The mutated positions are detailed in each node. Mitogenomes haplotypes were used and the following InDel positions were discarded: 16193.xC, 309.xC, 315.xC, 523-524del, 524.xC.

References: COL, PE, GB, NG, and SL: 1000 Genomes Project Consortium et al. *Nature* (2015), doi:10.1038/nature15393; PA: Simão et al. *Forensic Science International: Genetics* (2019), doi:10.1016/j.fsigen.2018.12.007; ANG: Barbieri et al. *PloS One* (2014), doi:10.1371/journal.pone.0099117.
